# Exploratory outcomes of the DHA WIN randomized controlled trial: Supplementing women with docosahexaenoic acid did not reduce the impact of neoadjuvant breast cancer chemotherapy on quality of life or exercise behaviour

**DOI:** 10.1371/journal.pone.0322178

**Published:** 2025-05-02

**Authors:** Claire M. Douglas, Marnie Newell, Susan Goruk, Kerry S. Courneya, Sunita Ghosh, Anil A. Joy, Jaqueline Munhoz, Catherine J. Field

**Affiliations:** 1 Department of Agricultural, Food and Nutritional Science, University of Alberta, Edmonton, Alberta, Canada; 2 Faculty of Kinesiology, Sport and Recreation, University of Alberta, Edmonton, Alberta, Canada; 3 Department of Oncology, University of Alberta, Edmonton, Alberta, Canada; 4 Department of Public Health Sciences, Henry Ford Hospital, Detroit, Michigan, United States of America; Universiti Monash Malaysia: Monash University Malaysia, MALAYSIA

## Abstract

Supplementation of omega-3 (n-3) polyunsaturated fatty acids has been associated with reduced side effects and improved quality of life (QoL) in breast cancer patients receiving chemotherapy. The current study reports secondary outcomes from the DHA WIN randomized controlled trial which was designed to evaluate docosahexaenoic acid (DHA) supplementation (4.4 g/day) in conjunction with six cycles of neoadjuvant chemotherapy (NAC) (3 weeks/cycle) in women with non-metastatic breast cancer (n = 49). The objective of the current study was to assess the effects of DHA supplementation on QoL and exercise behaviour in women undergoing NAC for breast cancer. Self-administered questionnaires were used to measure QoL and exercise behaviour before starting chemotherapy (baseline), before each chemotherapy cycle (exercise), and after completing chemotherapy. DHA supplementation did not significantly affect QoL, aerobic exercise volume or resistance training frequency during treatment. However, mean aerobic exercise volume was significantly lower at week 12 (-53.5 minutes/week; 95% CI, -100.5 to -6.3; p = 0.02) and week 18 (-70.8 minutes/week; 95% CI, -123.0 to -18.6; p = 0.01) compared to baseline. Mean resistance training frequency was lower at week 12 (-0.57 times/week; 95% CI, -1.0 to -0.13; p = 0.02) compared to baseline. Meeting exercise guidelines during chemotherapy was not associated with better QoL. In the current exploratory study, QoL and exercise decreased during treatment regardless of DHA supplementation, highlighting the need for supportive care and potential therapies that may mitigate these declines in breast cancer patients receiving NAC. Adequately powered studies are needed to determine if DHA supplementation improves these two indices of health. The trial is registered at ClinicalTrials.gov (NCT03831178).

## Introduction

An estimated 2.3 million women were diagnosed with breast cancer in 2022, making it the second most common cancer worldwide [1]. Neoadjuvant chemotherapy (NAC) is used to treat women with early-stage, operable breast cancer to improve surgical resection outcomes [2]. NAC is more frequently prescribed to patients with more aggressive subtypes including triple-negative breast cancer and HER2 + breast cancer [3,4]. Treatment for non-metastatic breast cancer frequently involves preoperative (neoadjuvant) systemic chemotherapy prior to surgical removal of the tumour [4]. Health-related quality of life (QoL) considers how disease and treatment affect an individual’s sense of overall function and well-being [5]. QoL has become an important outcome measure and factor in treatment decisions for NAC [6,7]. Chemotherapy treatment is reported to be associated with several physical and psychosocial side effects including nausea, vomiting, fatigue, impaired cognitive function and pain which influence QoL and exercise capacity [6–11]. All of these contribute to reduced treatment tolerability and worse clinical outcomes [12].

Supplementation of omega-3 (n-3) long chain polyunsaturated fatty acids to women receiving other chemotherapy regimens has been reported to result in reduction of some chemotherapy side effects including modulation of inflammatory profiles, reduction of gastrointestinal side effects, maintenance of skeletal muscle and improved neuronal recovery [13]. A recent review by Newell *et al*. (2021) concluded that supplementation of n-3 long chain polyunsaturated fatty acids in clinical cancer therapy improved overall QoL among patients with various types of cancer [14]. However, these studies did not report on women receiving NAC.

There are currently no specific dietary recommendations for long chain polyunsaturated fatty acids during cancer therapy or survivorship. Our group has demonstrated that feeding diets supplemented with the n-3 long chain polyunsaturated fatty acid, docosahexaenoic acid (DHA), reduces tumour growth and improves the efficacy of chemotherapy [15]. The effects on QoL or exercise capacity have not been studied. Although exercise has been demonstrated to be beneficial for relieving side-effects and improving QoL during chemotherapy [9], there are no clinical recommendations. However, based on the protective effects of exercise on mortality, the World Health Organization (WHO) recommends that cancer survivors do at least 150–300 minutes of moderate-intensity aerobic physical activity per week, as well as muscle strengthening activities two or more days per week [16].

The objective of the current manuscript was to assess the effects of DHA supplementation and NAC on self-reported QoL and exercise behaviour in women undergoing breast cancer treatment. These represent analyses of secondary outcomes from the Docosahexaenoic Acid for Women with Breast Cancer in the Neoadjuvant Setting (DHA WIN) randomized controlled trial [17].

## Materials and methods

### Setting and participants

The protocol for the DHA WIN RCT including inclusion and exclusion criteria has previously been published [17]. The trial included women with stage I-III non-metastatic breast cancer prescribed neoadjuvant chemotherapy. Participants were recruited by oncologists and clinical trial nurses at the Cross Cancer Institute (Edmonton, Alberta, Canada) and screened for eligibility. The DHA WIN RCT received Health Canada approval (#HC6–24-c220167) and full ethical approval from the Health Research Ethics Board of Alberta - Cancer Committee (HREBA.CC-18–0381) [17]. Written informed consent was obtained prior to individuals’ involvement in the study. The target sample size of 26 participants per treatment arm was determined based on the primary outcome of the trial (change in the Ki67 index in the tumour).

### Study design and procedures

The DHA WIN trial was a two-arm, double-blind, phase II RCT designed to investigate the effects of DHA supplementation concomitant with neoadjuvant chemotherapy in women with non-metastatic breast cancer [17]. All women received standard-of-care chemotherapy, which was one of two docetaxel-based neoadjuvant chemotherapy regimens that were used in this population. Each regimen consisted of six cycles of chemotherapy that were administered in 3-week intervals (for a total of 18 weeks). QoL questionnaires were completed at baseline (week 0) and the end of cycle 6 (week 18). Exercise questionnaires were completed at the start of each 3-week cycle, and the end of cycle 6 (week 18). Participants were given paper copies of the questionnaires to complete during clinic visits.

### Randomization

Block randomization occurred as previously described [17]. Briefly, a biostatistician generated a patient randomization list and randomized bottle numbers using covariate-adaptive randomization. The randomized bottle list was provided to DSM Nutritional Products for labelling purposes, as well as the unblinded Clinical Trials Coordinator (CTC) and pharmacist. The REDCap database was used to allocate participants to a study arm and provide a unique study identifier by the study coordinator. Pharmacy staff assigned bottle numbers based on treatment arm on the first day of each chemotherapy cycle. The bottle ID was then entered into the REDCap database by the study coordinator. Participants, pathologists, physicians and researchers were all blinded throughout the trial. The CTC, statistician and pharmacist were unblinded. DHA and placebo supplements were identical in size, shape, colour, texture and bottles.

### Intervention

Participants were randomized to receive 4.4 g/day of DHA or a placebo supplement [17]. The DHA group received eleven 1 g DHA-enriched, algae-sourced triglyceride oil capsules (life’sDHA S40-O400), while the placebo group received eleven 1 g capsules of a corn/soy oil blend per day (DSM Nutritional Products, Columbia, Maryland, USA). The placebo supplement contained equal amounts of polyunsaturated fatty acids as the DHA supplement, in the form of linoleic acid. Participants were instructed to orally consume the capsules at any time throughout the day, with or without food. The intervention began at the start of the first cycle of chemotherapy and continued throughout chemotherapy treatment. Compliance was determined by a review of the patient dosing diary and the recorded number of any remaining capsules returned at the end of the study.

### Assessment of QoL and exercise behaviour

Questionnaires to record QoL and exercise behaviour were given to participants before starting chemotherapy (baseline), before each chemotherapy cycle (exercise), and after completing chemotherapy. Participants were instructed on how to fill these out, but there was not a method used to ensure accuracy of the responses. Cancer-specific QoL and fatigue were assessed using the Functional Assessment of Cancer Therapy (FACT) – General (FACT-G) [18,19], FACT – Breast (FACT-B) [20,21], FACT – Taxane [22,23], FACT – Endocrine Symptoms (FACT-ES) [24,25] and the Functional Assessment of Chronic Illness Therapy (FACIT) – Fatigue (FACIT-Fatigue) scales [26,27]. Higher scores indicate better QoL (and less fatigue) among the aforementioned scales. Psychosocial functioning was assessed using the Perceived Stress Scale (PSS) (with higher scores indicating greater stress) [28], the Fordyce Emotions Questionnaire (with higher scores indicating greater happiness) [29], and modified versions of the Center for Epidemiologic Studies-Depression Scale (CES-D) (with higher scores indicating greater depressive symptomology) [30] and the State-Trait Anxiety Inventory (STAI) (with higher scores indicating greater anxiety) [31].

An adapted version of the Godin Leisure-Time Exercise Questionnaire was used to collect information on the frequency and average duration of light, moderate and vigorous aerobic exercise as well as strength/resistance training per week [32,33]. Average weekly aerobic exercise was determined by adding the average minutes of moderate aerobic exercise to two times the average minutes of vigorous aerobic exercise. Aerobic exercise volume and resistance training frequency were averaged (excluding baseline levels) and used to categorize participants on the basis of meeting WHO’s 2020 exercise recommendations (≥ 150 minutes/week aerobic exercise and ≥ 2 times/week resistance training) [16].

### Statistical analyses

Descriptive statistics including means (SD) and proportions (frequencies) were used to describe continuous and categorical variables, respectively. Baseline values were compared between groups using the independent t-test for continuous variables and the chi-squared test for categorical variables. Missing samples were completely at random and were therefore excluded from analysis. One participant in the placebo group had no available QoL data at the end of treatment. The legends of Figures 2 and 3 show the fewest number of participants with data, as this number varied at each time point. Analysis of covariance was used to examine the effects of DHA treatment on QoL scores as well as associations between exercise classifications and QoL scores. Generalized estimating equations (GEE) method was used to assess the effects of time and DHA treatment on the average volume of aerobic exercise and average frequency of resistance training. GEE method is used to analyze correlated data and to assess the between and within subject variability for repeated measures data, and provides unbiased parameter estimates and robust standard errors. Time point comparisons were made to the baseline levels of exercise. Both unadjusted and adjusted analyses were conducted. For the adjusted analysis the GEE models were adjusted for age, BMI and baseline value of the outcome. The post hoc analyses were not adjusted for multiple comparisons. Therefore, the results of this study should be interpreted as exploratory. Statistical significance was defined as a two-sided p-value < 0.05. Statistical analyses and creation of figures were conducted using SPSS (V27.0, IBM Corporation, Armonk, New York, USA).

**Fig 1:**
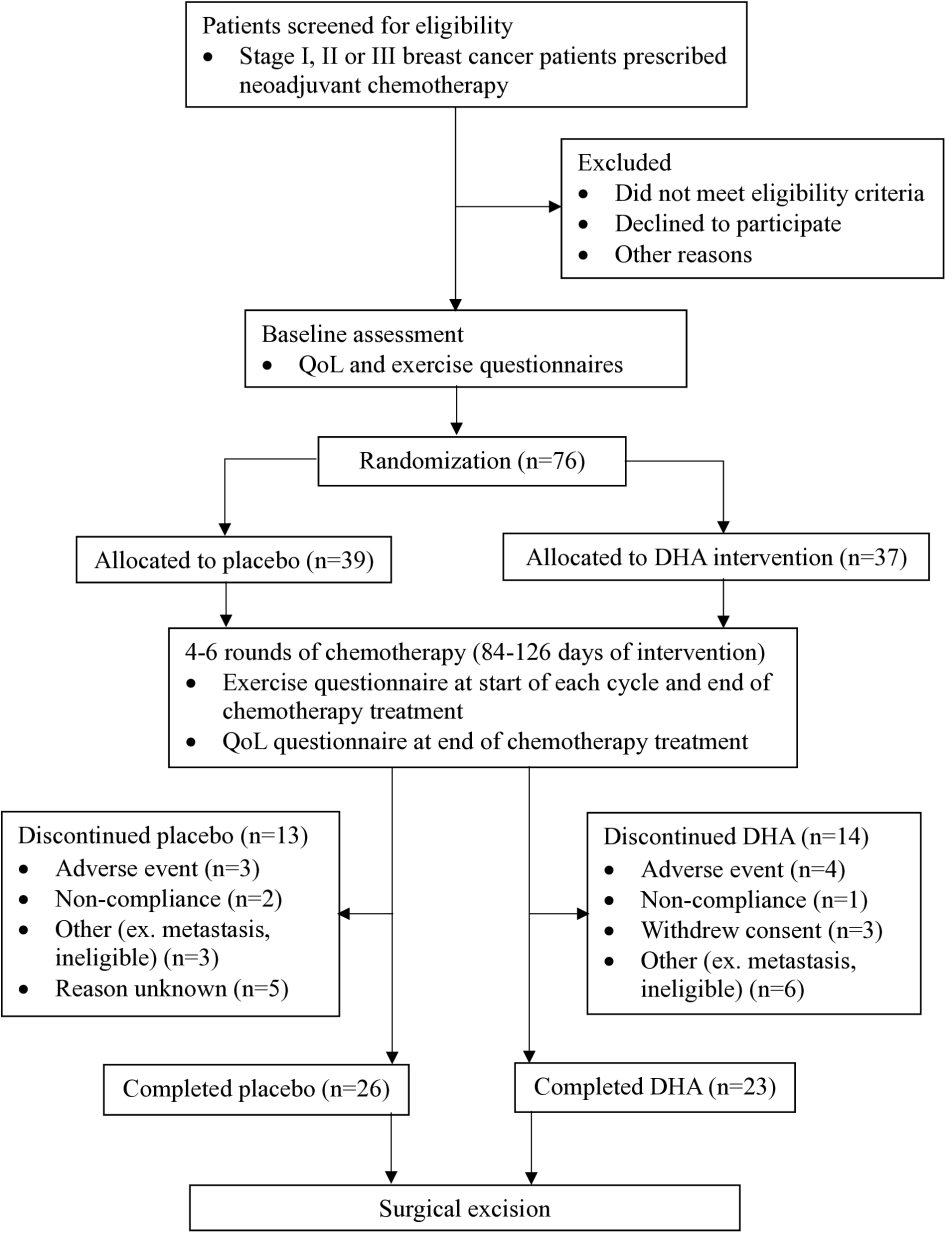
Flow of participants through the DHA WIN RCT. Abbreviations: Docosahexaenoic acid (DHA), Docosahexaenoic acid for Women with Breast Cancer in the Neoadjuvant Setting (DHA WIN), randomized controlled trial (RCT).

**Fig 2:**
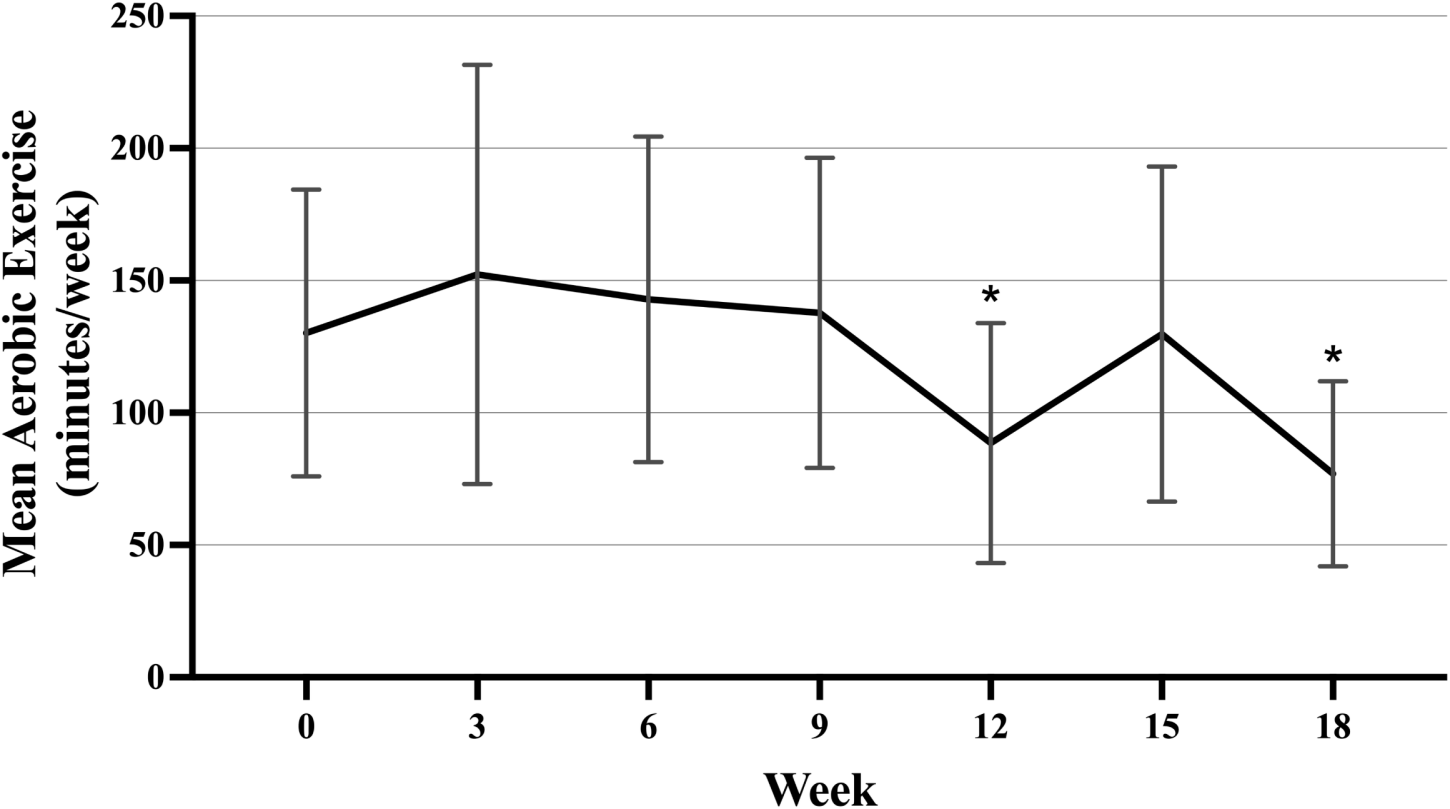
Weekly aerobic exercise volume over time for the DHA and placebo groups combined (n **= 42).** Error bars represent 95% confidence intervals. Generalized estimating equations were used to test statistical significance of differences within and between treatment groups. Models were adjusted for age, BMI and baseline aerobic exercise level. Each week was compared to baseline using unadjusted multiple comparisons. *Indicates statistically significantly different from baseline at p ≤ 0.03. Week 0 indicates baseline. Week 18 indicates the end of chemotherapy. Abbreviations: Docosahexaenoic acid (DHA), body mass index (BMI).

**Fig 3:**
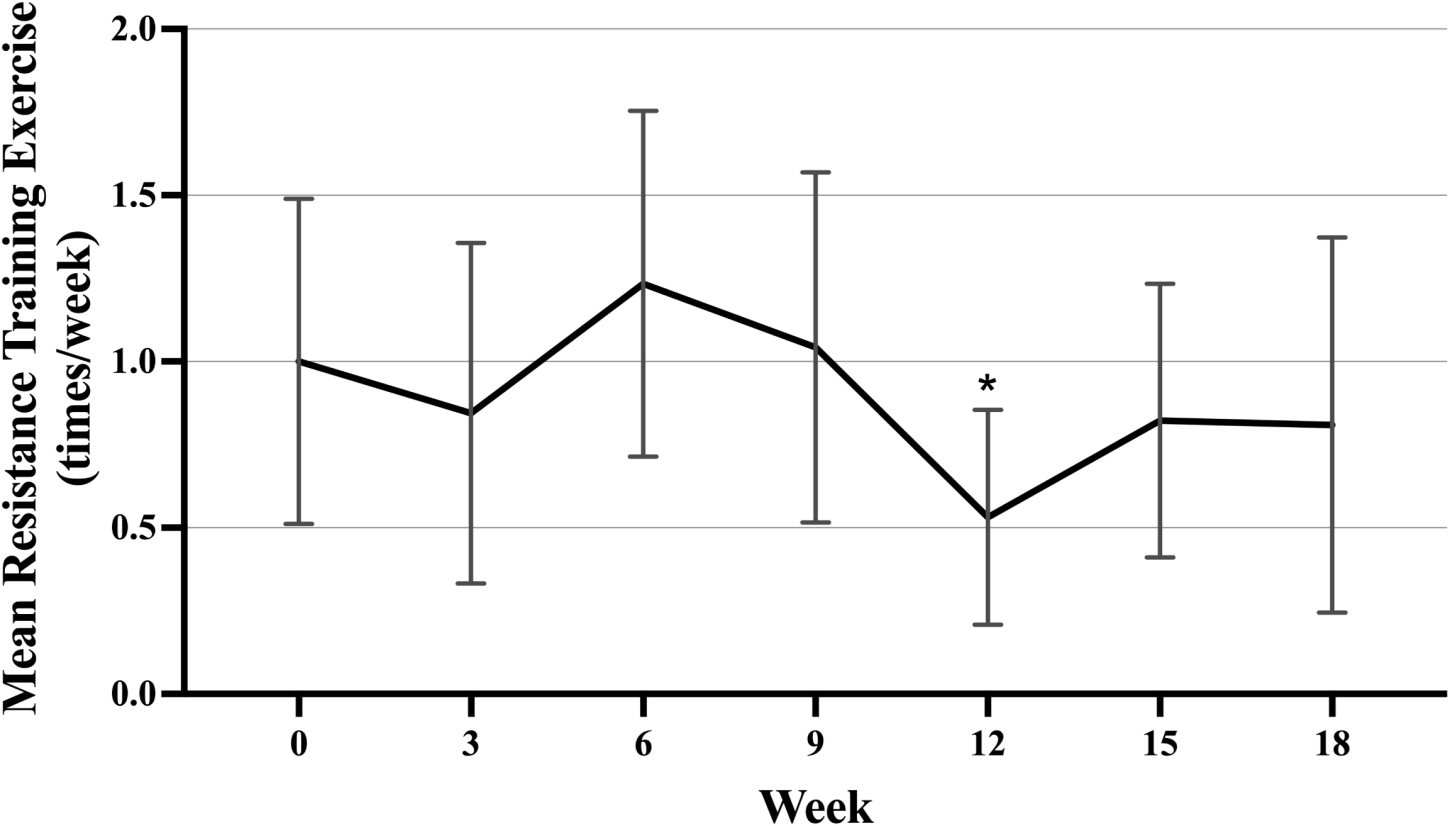
Weekly resistance training frequency over time for the DHA and placebo groups combined (n **= 42).** Error bars represent 95% confidence intervals. Generalized estimating equations were used to test statistical significance of differences within and between treatment groups. Models were adjusted for age, BMI and baseline resistance training exercise frequency. Each week was compared to baseline using unadjusted multiple comparisons. *Indicates statistically significantly different from baseline at p ≤ 0.03. Week 0 indicates baseline. Week 18 indicates the end of cycle 6. Abbreviations: Docosahexaenoic acid (DHA), body mass index (BMI).

## Results

### Participant flow and baseline characteristics

Recruitment of participants took place between September 27, 2019 and May 30, 2022. The trial ended in December 2022 when the sample size was obtained. Follow-up assessments are scheduled for 3, 5 and 10 years to assess long term outcomes. Seventy-six participants were randomized to the placebo or DHA intervention, of which 49 (65%) completed the DHA WIN trial (Fig 1). Adherence to the intervention was 78% for the placebo group and 86% for the DHA group (p = 0.26). At baseline, the participant demographics and clinical characteristics did not differ between groups (Table 1).

**Table 1:** Participant demographic and clinical characteristics.

	Total (n = 49)	Placebo (n = 26)	DHA (n = 23)	p-value
**Age (years)** ^*^	50.8 ± 10.7	51.2 ± 12.0	50.4 ± 9.3	0.80
**BMI (kg/m** ^**2**^ **)** ^*^	28.8 ± 6.7	27.5 ± 6.0	30.3 ± 7.3	
Underweight (<18.5)^†^	1 (2.0)	1 (3.8)	0 (0.0)	0.60
Healthy weight (18.5–24.9)^†^	13 (26.5)	8 (30.8)	5 (21.7)	
Overweight (25–29.9)^†^	18 (36.7)	10 (38.5)	8 (34.8)	
Obese (≥30)^†^	17 (34.7)	7 (26.9)	10 (43.5)	
**Ethnicity** ^†^				
Not Hispanic or Latino	32 (65.3)	17 (65.4)	15 (65.2)	0.89
Asian	8 (16.3)	5 (19.2)	3 (13.0)	
Black or African American	4 (8.2)	2 (7.7)	2 (8.7)	
American Indian or Alaska Native	5 (10.2)	2 (7.7)	3 (13.0)	
**Menopausal status** ^†^				
No	25 (51.0)	13 (50.0)	12 (52.2)	0.31
Yes	22 (44.9)	13 (50.0)	9 (39.1)	
Missing	2 (4.1)	0 (0.0)	2 (8.7)	
**Age at menarche (years)** ^*^	12.8 ± 1.5	12.7 ± 1.7	12.8 ± 1.3	0.82
Missing^†^	4 (8.2)	2 (7.7)	2 (8.7)	
**Diabetes** ^†^				
No	47 (95.9)	26 (100.0)	21 (91.3)	0.22
Yes	2 (4.1)	0 (0.0)	2 (8.7)	
**Smokers** ^†^				
No	41 (83.7)	21 (80.8)	20 (87.0)	0.42
Yes	8 (16.3)	5 (19.2)	3 (13.0)	
**Use of recreational drugs** ^†^				
No	44 (89.8)	24 (92.3)	20 (87.0)	0.44
Yes	5 (10.2)	2 (7.7)	3 (13.0)	
**ECOG** ^†^ ^,^ ^‡^				
**Baseline**				
0	44 (89.8)	24 (92.3)	20 (87.0)	0.05
1	2 (4.1)	2 (7.7)	0 (0.0)	
Missing	3 (6.1)	0 (0.0)	3 (13.0)	
**End of treatment**				
0	34 (69.4)	21 (80.8)	13 (56.5)	0.36
1	11 (22.4)	3 (11.5)	8 (34.8)	
2	2 (4.1)	1 (3.8)	1 (4.3)	
Missing	2 (4.1)	1 (3.8)	1 (4.3)	
**Total recruited**	76	39	37	0.43
Completed^†^	49 (64.5)	26 (66.7)	23 (62.2)	
Withdrawn^†^	27 (35.5)	13 (33.3)	14 (37.8)	
**Histology** ^†^ ^,^ ^§^				
HER2+	25 (51.0)	14 (53.8)	11 (47.8)	0.87
TNBC	12 (24.5)	5 (19.2)	7 (30.4)	
Luminal A	10 (20.4)	6 (23.1)	4 (17.4)	
Luminal B	2 (4.1)	1 (3.8)	1 (4.3)	
**Estrogen receptor status** ^†^				
Positive	26 (53.1)	16 (61.5)	10 (43.5)	0.21
Negative	23 (46.9)	10 (38.5)	13 (56.5)	
**Progesterone receptor status** ^†^				
Positive	17 (34.7)	11 (42.3)	6 (26.1)	0.24
Negative	31 (63.3)	14 (53.8)	17 (73.9)	
Missing	1 (2.0)	1 (3.8)	0 (0.0)	
**HER2 Status** ^†^				
Positive	23 (46.9)	12 (46.2)	11 (47.8)	1.00
Negative	25 (51.0)	13 (50.0)	12 (52.2)	
Missing	1 (2.0)	1 (3.8)	0 (0.0)	
**Disease Stage** ^†^				
IIA	13 (26.5)	6 (23.1)	7 (30.4)	0.63
IIB	10 (20.4)	4 (15.4)	6 (26.1)	
IIIA	14 (28.6)	9 (34.6)	5 (21.7)	
IIIB	4 (8.2)	2 (7.7)	2 (8.7)	
IIIC	1 (2.0)	0 (0.0)	1 (4.3)	
Missing	7 (14.3)	5 (19.2)	2 (8.7)	
**Tumour Size** ^†^				
T1	1 (2.0)	1 (3.8)	0 (0.0)	0.92
T2	27 (55.1)	15 (57.7)	12 (52.2)	
T3	11 (22.4)	5 (19.2)	6 (26.1)	
T4	5 (10.2)	3 (11.5)	2 (8.7)	
Missing	5 (10.2)	2 (7.7)	3 (13.0)	
**Axillary Node Status** ^†^				
N0	12 (24.5)	6 (23.1)	6 (26.1)	1.00
N1	21 (42.9)	11 (42.3)	10 (43.5)	
N2	5 (10.2)	3 (11.5)	2 (8.7)	
N3	2 (4.1)	1 (3.8)	1 (4.3)	
Missing	9 (18.4)	5 (19.2)	4 (17.4)	

*Mean ± SD;

† Count (percentage of total or given group);

‡ 0 = Fully active, able to carry on all pre-disease performance without restriction; 1 = Restricted in physically strenuous activity but ambulatory and able to carry out work of a light or sedentary nature, e.g., light housework, office work; 2 = Ambulatory and capable of all selfcare but unable to carry out any work activities, up and about more than 50% of waking hours;

§ HER2+ (HER2+, with or without ER+, and/or PR+), TNBC (ER-, PR-, HER2-), Luminal A (ER + and/or PR + , HER2-), Luminal B (ER + and/or PR + , HER2-). One patient in the DHA group reported a history of ethanol abuse. Abbreviations: Body mass index (BMI), human epidermal growth factor receptor 2 (HER2), triple negative breast cancer (TNBC), Eastern Cooperative Oncology Group (ECOG).

### Changes in QoL of DHA and placebo groups

Table 2 illustrates the QoL scores of participants at baseline (week 0) and the end of chemotherapy (week 18). There was no significant difference in the adjusted mean change between randomized groups for any QoL indicator (Table 2).

**Table 2:** Quality of life scores at baseline (week 0) and end of chemotherapy (week 18) in the DHA and placebo groups.

Outcome	Week 0, mean (SD)	Week 18, mean (SD)	Mean change, mean (95% CI)	Adjusted between group difference in mean change, mean (95% CI); *p*
**FACT-G total score**				
Placebo	85.2 (14.1)	79.4 (16.0)	-5.9 (-14.9 to 3.1)	+2.5 (-4.4 to 9.4); *p* = 0.47
DHA	88.9 (14.0)	82.2 (17.0)	-6.7 (-16.0 to 2.7)	
**Physical WB**				
Placebo	24.5 (4.4)	21.1 (6.8)	-3.4 (-6.7 to -0.1)	+1.3 (-1.6 to 4.2); *p* = 0.38
DHA	24.6 (4.4)	21.8 (5.0)	-2.8 (-5.6 to 0.03)	
**Social/family WB**				
Placebo	23.3 (5.1)	21.6 (5.4)	-1.7 (-4.7 to 1.3)	+0.5 (-1.8 to 2.7); *p* = 0.68
DHA	24.8 (4.2)	23.2 (4.8)	-1.6 (-4.3 to 1.1)	
**Emotional WB**				
Placebo	17.1 (5.1)	19.1 (3.9)	+2.0 (-0.5 to 4.6)	-0.1 (-1.9 to 1.8); *p* = 0.95
DHA	17.6 (4.8)	18.8 (4.2)	+1.2 (-1.5 to 3.9)	
**Functional WB**				
Placebo	20.1 (5.1)	18.0 (5.1)	-2.1 (-5.0 to 0.8)	+0.1 (-2.8 to 3.1); *p* = 0.94
DHA	21.9 (5.9)	18.4 (5.8)	-3.5 (-7.0 to 0.0)	
**FACT-B Total Score**				
Placebo	115.7 (18.0)	106.9 (19.9)	-8.8 (-19.8 to 2.2)	+2.4 (-6.3 to 11.0); *p* = 0.58
DHA	118.6 (17.0)	108.5 (21.8)	-10.2 (-21.9 to 1.6)	
**FACT-B TOI**				
Placebo	75.8 (11.2)	66.3 (13.7)	-9.5 (-16.6 to -2.3)	+1.7 (-4.9 to 8.3); *p* = 0.60
DHA	76.2 (12.5)	66.5 (15.0)	-9.8 (-18.0 to -1.5)	
**BC Subscale score**				
Placebo	31.2 (4.4)	27.8 (5.5)	-3.3 (-6.1 to -0.5)	-0.7 (-4.0 to 2.6); *p* = 0.65
DHA	29.7 (5.9)	26.2 (6.7)	-3.5 (-7.3 to 0.3)	
**FACT-Tax Total Score**				
Placebo	147.8 (15.1)	135.1 (21.4)	-12.7 (-23.6 to -1.8)	+1.3 (-8.0 to 10.7); *p* = 0.77
DHA	150.2 (18.4)	136.9 (21.4)	-13.3 (-25.3 to -1.3)	
**FACT-Tax TOI**				
Placebo	105.9 (12.2)	94.5 (17.3)	-11.3 (-20.0 to -2.6)	+1.0 (-7.0 to 9.0); *p* = 0.80
DHA	107.8 (14.8)	94.8 (14.8)	-12.9 (-21.8 to -4.1)	
**Taxane subscale score**				
Placebo	60.9 (6.5)	56.0 (9.3)	-4.9 (-9.5 to -0.3)	-1.1 (-5.9 to 3.7); *p* = 0.65
DHA	61.3 (6.0)	54.6 (7.1)	-6.6 (-10.6 to -2.7)	
**FACT-ES Total Score**				
Placebo	155.3 (17.0)	144.6 (21.1)	-10.7 (-22.0 to 0.6)	+2.7 (-6.3 to 11.6); *p* = 0.55
DHA	154.9 (21.5)	145.7 (22.9)	-9.2 (-22.5 to 4.2)	
**ES subscale score**				
Placebo	69.6 (4.9)	65.4 (7.7)	-4.2 (-7.8 to -0.5)	-0.3 (-4.5 to 3.8); *p* = 0.87
DHA	66.0 (9.4)	63.5 (8.0)	-2.5 (-7.8 to 2.8)	
**Fatigue subscale score**				
Placebo	43.1 (8.2)	38.0 (10.9)	-5.2 (-10.6 to 0.2)	-0.1 (-5.4 to 5.2); *p* = 0.98
DHA	44.3 (11.1)	38.0 (10.7)	-6.3 (-12.9 to 0.3)	
**PSS score**				
Placebo	20.2 (8.5)	19.2 (8.7)	-1.0 (-5.9 to 3.8)	-1.7 (-5.6 to 2.2); *p* = 0.38
DHA	19.7 (7.2)	18.0 (9.2)	-1.7 (-6.6 to 3.2)	
**CES-D score**				
Placebo	7.3 (4.3)	7.8 (3.9)	+0.5 (-1.8 to 2.8)	-0.8 (-2.5 to 0.9); *p* = 0.35
DHA	7.0 (3.2)	7.3 (4.0)	+0.3 (-1.9 to 2.4)	
**STAI score**				
Placebo	20.8 (6.1)	18.9 (6.0)	-1.9 (-5.3 to 1.5)	-0.5 (-3.4 to 2.4); *p* = 0.72
DHA	20.4 (7.0)	18.7 (6.6)	-1.7 (-5.8 to 2.4)	
**Fordyce Emotions Combination Score**				
Placebo	57.6 (22.4)	68.1 (17.4)	+10.5 (-1.2 to 22.2)	-5.0 (-15.1 to 5.1); *p* = 0.32
DHA	59.4 (24.2)	63.5 (21.8)	+4.1 (-10.2 to 18.5)	

Analysis of covariance was used to test statistical significance of differences in mean change between DHA (n = 22) and placebo (n = 20) groups. Difference in mean change was adjusted for age, BMI, and baseline value of the outcome. Abbreviations: Docosahexaenoic acid (DHA), Functional Assessment of Cancer Therapy-General (FACT-G), well-being (WB), FACT-Breast (FACT-B), Trial Outcome Index (TOI), breast cancer (BC), endocrine symptoms (ES), Perceived Stress Scale (PSS), Center for Epidemiologic Studies-Depression (CES-D), State-Trait Anxiety Inventory (STAI), body mass index (BMI).

### Comparing exercise behaviour within and between treatment groups

Dietary treatment did not significantly affect the change in mean weekly aerobic exercise volume (p-interaction = 0.53) or resistance training frequency (p-interaction = 0.15). Therefore, treatment groups were combined to assess changes over time. Overall, mean aerobic exercise was lower at week 12 (-53.5 minutes/week; 95% CI, -100.5 to -6.3; p = 0.02) and week 18 (-70.8 minutes/week; 95% CI, -123.0 to -18.6; p = 0.01) compared to baseline (Fig 2). Mean resistance training frequency was lower at week 12 (-0.57 times/week; 95% CI, -1.0 to -0.13; p = 0.02) compared to baseline (Fig 3).

### Changes in QoL grouped by WHO’s exercise recommendations

Fourteen participants (29%) met WHO’s aerobic exercise recommendation, while 35 (71%) did not. Seven participants (14%) met WHO’s resistance training recommendation, while 42 (86%) did not. Differences in the adjusted mean change between the two groups were not statistically significant for any QoL outcome (Tables 3 and 4).

**Table 3:** Quality of life scores at baseline (week 0) and the end of chemotherapy (week 18) in participants that met (≥ 150 minutes/week) or did not meet (< 150 minutes/week) WHO’s aerobic exercise recommendation on average during chemotherapy.

Outcome	Week 0, mean (SD)	Week 18, mean (SD)	Mean change, mean (95% CI)	Adjusted between group difference in mean change, mean (95% CI); *p*
**FACT-G total score**				
< 150 min/week	86.0 (13.7)	78.6 (17.6)	-7.4 (-15.3 to 0.5)	+2.7 (-4.6 to 9.9); *p* = 0.46
≥ 150 min/week	89.9 (15.1)	85.7 (12.3)	-4.3 (-15.1 to 6.6)	
**Physical WB**				
< 150 min/week	24.0 (4.8)	20.8 (6.3)	-3.3 (-6.0 to -0.6)	+0.2 (-3.0 to 3.4); *p* = 0.89
≥ 150 min/week	25.9 (2.5)	23.1 (5.0)	-2.8 (-5.9 to 0.3)	
**Social/family WB**				
< 150 min/week	24.2 (4.1)	22.5 (4.6)	-1.7 (-3.8 to 0.4)	-0.1 (-2.4 to 2.2); *p* = 0.93
≥ 150 min/week	23.5 (6.3)	22.1 (6.4)	-1.4 (-6.4 to 3.5)	
**Emotional WB**				
< 150 min/week	17.0 (5.4)	18.6 (4.4)	+1.5 (-0.9 to 3.9)	+0.4 (-1.7 to 2.4); *p* = 0.73
≥ 150 min/week	18.1 (3.5)	19.9 (2.4)	+1.8 (-0.5 to 4.1)	
**Functional WB**				
< 150 min/week	20.5 (5.8)	17.1 (5.7)	-3.4 (-6.2 to -0.6)	2.4 (-0.7 to 5.5); *p *= 0.12
≥ 150 min/week	22.1 (4.6)	20.7 (3.5)	-1.4 (-4.6 to 1.8)	
**FACT-B Total Score**				
< 150 min/week	115.0 (16.5)	105.6 (21.7)	-9.4 (-18.8 to 0.03)	-1.3 (-10.8 to 8.1); *p* = 0.78
≥ 150 min/week	122.6 (19.1)	112.2 (17.8)	-10.4 (-25.0 to 4.3)	
**FACT-B TOI**				
< 150 min/week	74.2 (11.6)	64.6 (14.9)	-9.6 (-16.1 to -3.1)	-1.4 (-8.7 to 5.9); *p* = 0.71
≥ 150 min/week	80.8 (10.9)	70.3 (11.8)	-10.4 (-19.5 to -1.4)	
**BC Subscale score**				
< 150 min/week	29.6 (5.1)	27.3 (6.0)	-2.3 (-5.0 to 0.4)	-3.2 (-6.9 to 0.5); *p* = 0.08
≥ 150 min/week	32.6 (4.9)	26.6 (6.5)	-6.1 (-10.7 to -1.5)	
**FACT-Tax Total Score**				
< 150 min/week	147.2 (17.2)	133.4 (23.2)	-13.8 (-23.9 to -3.7)	0.5 (-9.7 to 10.7); *p* = 0.92
≥ 150 min/week	153.6 (15.0)	141.7 (14.7)	-11.9 (-23.7 to -0.1)	
**FACT-Tax TOI**				
< 150 min/week	104.9 (14.9)	92.5 (17.6)	-12.4 (-20.4 to -4.5)	0.1 (-8.6 to 8.9); *p* = 0.98
≥ 150 min/week	111.7 (6.9)	99.8 (10.4)	-12.0 (-19.0 to -4.9)	
**Taxane subscale score**				
< 150 min/week	60.0 (7.1)	55.1 (8.6)	-4.9 (-8.8 to -1.1)	-1.9 (-7.3 to 3.6); *p* = 0.49
≥ 150 min/week	63.6 (0.8)	56.0 (7.7)	-7.6 (-12.1 to -3.2)	
**FACT-ES Total Score**				
< 150 min/week	153.1 (19.9)	143.2 (22.7)	-9.9 (-20.4 to 0.7)	-0.2 (-10.0 to 9.7); *p* = 0.97
≥ 150 min/week	160.3 (16.7)	149.6 (19.4)	-10.7 (-25.1 to 3.8)	
**ES subscale score**				
< 150 min/week	66.7 (8.4)	64.8 (7.5)	-1.9 (-5.8 to 2.0)	-2.3 (-6.8 to 2.2); *p* = 0.31
≥ 150 min/week	70.8 (3.6)	64.0 (8.9)	-6.8 (-12.1 to -1.5)	
**Fatigue subscale score**				
< 150 min/week	42.4 (10.2)	36.4 (10.5)	-6.0 (-11.0 to -0.9)	1.4 (-4.5 to 7.2); *p* = 0.64
≥ 150 min/week	46.9 (7.0)	41.7 (10.7)	-5.2 (-12.3 to 1.8)	
**PSS score**				
< 150 min/week	20.0 (8.0)	19.5 (8.9)	-0.4 (-4.5 to 3.7)	-2.1 (-6.3 to 2.2); *p* = 0.33
≥ 150 min/week	19.9 (7.7)	16.4 (8.6)	-3.5 (-9.8 to 2.8)	
**CES-D score**				
< 150 min/week	7.4 (3.3)	8.1 (3.7)	+0.6 (-1.1 to 2.4)	-0.5 (-2.4 to 1.4); *p* = 0.58
≥ 150 min/week	6.6 (4.8)	6.5 (4.2)	-0.1 (-3.6 to 3.4)	
**STAI score**				
< 150 min/week	21.3 (6.7)	19.9 (6.1)	-1.4 (-4.5 to 1.7)	-2.0 (-5.2 to 1.2); *p* = 0.22
≥ 150 min/week	18.8 (5.7)	16.1 (5.7)	-2.7 (-7.1 to 1.7)	
**Fordyce Emotions Combination Score**				
< 150 min/week	52.7 (23.7)	61.0 (20.4)	+8.3 (-2.8 to 19.4)	7.3 (-4.1 to 18.6); *p* = 0.20
≥ 150 min/week	71.8 (14.4)	77.6 (11.1)	+5.8 (-4.4 to 16.1)	

Analysis of covariance was used to test statistical significance of differences in mean change between groups that met (n = 14) or did not meet (n = 35) WHO’s aerobic exercise recommendation. Difference in mean change was adjusted for age, BMI, and baseline value of the outcome. Abbreviations: World Health Organization (WHO), Functional Assessment of Cancer Therapy-General (FACT-G), well-being (WB), FACT-Breast (FACT-B), Trial Outcome Index (TOI), breast cancer (BC), endocrine symptoms (ES), Perceived Stress Scale (PSS), Center for Epidemiologic Studies-Depression (CES-D), State-Trait Anxiety Inventory (STAI), body mass index (BMI).

**Table 4:** Quality of life scores at baseline (week 0) and the end of chemotherapy (week 18) in participants that met (≥ 2 times/week) or did not meet (< 2 times/week) WHO’s resistance training exercise recommendation on average during chemotherapy.

Outcome	Week 0, mean (SD)	Week 18, mean (SD)	Mean change, mean (95% CI)	Adjusted between group difference in mean change, mean (95% CI); *p*
**FACT-G total score**				
< 2 times/week	86.4 (14.5)	79.4 (16.9)	-6.9 (-14.1 to 0.2)	+2.5 (-6.9 to 11.9); *p* = 0.60
≥ 2 times/week	92.0 (10.2)	88.0 (11.1)	-3.9 (-17.1 to 9.2)	
**Physical WB**				
< 2 times/week	24.3 (4.5)	21.1 (5.9)	-3.2 (-5.5 to -0.8)	0.4 (-3.6 to 4.5); *p* = 0.83
≥ 2 times/week	26.5 (2.2)	23.6 (6.2)	-2.9 (-8.3 to 2.5)	
**Social/family WB**				
< 2 times/week	24.1 (4.9)	22.3 (5.3)	-1.7 (-4.0 to 0.5)	0.6 (-2.3 to 3.5); *p* = 0.69
≥ 2 times/week	23.2 (3.8)	22.3 (4.6)	-0.9 (-5.8 to 4.0)	
**Emotional WB**				
< 2 times/week	16.9 (4.9)	18.6 (4.0)	+1.7 (-0.3 to 3.7)	0.6 (-2.0 to 3.3); *p* = 0.63
≥ 2 times/week	19.9 (4.7)	20.9 (3.1)	+1.0 (-3.7 to 5.7)	
**Functional WB**				
< 2 times/week	20.7 (5.7)	17.6 (5.5)	-3.1 (0.6 to 5.6)	2.6 (-1.2 to 6.4); *p* = 0.17
≥ 2 times/week	22.4 (4.6)	21.3 (3.1)	-1.1 (-3.5 to 5.7)	
**FACT-B Total Score**				
< 2 times/week	116.1 (17.6)	106.2 (21.0)	-9.9 (-18.5 to -1.3)	0.5 (-11.9 to 12.8); *p* = 0.94
≥ 2 times/week	123.8 (15.2)	115.5 (17.5)	-8.3 (-28.5 to 11.8)	
**FACT-B TOI**				
< 2 times/week	75.1 (11.8)	65.3 (14.3)	-9.7 (-15.6 to -3.9)	1.8 (-7.1 to 10.7); *p* = 0.69
≥ 2 times/week	81.4 (10.4)	72.3 (12.3)	-9.1 (-22.4 to 4.2)	
**BC Subscale score**				
< 2 times/week	30.1 (5.1)	27.0 (6.0)	-3.1 (-5.5 to -0.6)	-0.9 (-5.5 to 3.7); *p* = 0.70
≥ 2 times/week	32.6 (5.7)	27.4 (7.1)	-5.1 (-12.6 to 2.4)	
**FACT-Tax Total Score**				
< 2 times/week	147.9 (17.3)	135.1 (21.9)	-12.9 (-21.7 to -4.0)	-1.0 (-14.3 to 12.3); *p* = 0.88
≥ 2 times/week	156.0 (10.2)	140.9 (16.5)	-15.1 (-32.2 to 2.0)	
**FACT-Tax TOI**				
< 2 times/week	106.5 (13.3)	94.1 (16.6)	-12.3 (-19.0 to -5.6)	1.8 (-8.7 to 12.3); *p* = 0.73
≥ 2 times/week	108.7 (15.2)	97.7 (12.6)	-11.0 (-27.2 to 5.3)	
**Taxane subscale score**				
< 2 times/week	61.3 (5.2)	55.8 (8.3)	-5.5 (-8.6 to -2.4)	-2.5 (-9.1 to 4.1); *p* = 0.45
≥ 2 times/week	59.9 (11.0)	52.9 (8.4)	-7.0 (-18.3 to 4.3)	
**FACT-ES Total Score**				
< 2 times/week	153.7 (19.7)	143.9 (21.7)	-9.8 (-19.1 to -0.5)	-0.8 (-13.7 to 12.2); *p* = 0.90
≥ 2 times/week	164.6 (12.1)	152.3 (22.3)	-12.3 (-34.8 to 10.2)	
**ES subscale score**				
< 2 times/week	67.2 (7.8)	64.6 (7.2)	-2.6 (-6.0 to 0.7)	-1.4 (-7.1 to 4.3); *p* = 0.63
≥ 2 times/week	71.8 (3.4)	64.3 (11.4)	-7.6 (-17.4 to 2.2)	
**Fatigue subscale score**				
< 2 times/week	43.9 (9.5)	37.7 (10.5)	-6.2 (-10.6 to -1.8)	2.3 (-4.9 to 9.5); *p *= 0.52
≥ 2 times/week	42.6 (10.5)	39.7 (12.8)	-2.9 (-16.5 to 10.7)	
**PSS score**				
< 2 times/week	20.0 (7.7)	18.3 (9.2)	-1.7 (-5.4 to 2.0)	2.9 (-2.4 to 8.1); *p* = 0.28
≥ 2 times/week	19.6 (9.1)	20.3 (6.9)	+0.7 (-8.7 to 10.1)	
**CES-D score**				
< 2 times/week	7.5 (3.8)	7.6 (3.7)	+0.1 (-1.6 to 1.8)	1.6 (-0.7 to 4.0); *p* = 0.17
≥ 2 times/week	5.3 (3.4)	7.4 (5.0)	+2.1 (-2.8 to 7.1)	
**STAI score**				
< 2 times/week	21.0 (6.6)	18.9 (6.1)	-2.2 (-5.0 to 0.7)	1.4 (-2.7 to 5.4); *p* = 0.50
≥ 2 times/week	18.2 (5.2)	18.4 (7.3)	+0.2 (-7.2 to 7.6)	
**Fordyce Emotions Combination Score**				
< 2 times/week	56.6 (23.6)	63.8 (20.3)	+7.2 (-2.8 to 17.2)	8.1 (-6.4 to 22.6); *p* = 0.27
≥ 2 times/week	70.8 (13.7)	77.4 (9.7)	+6.5 (-7.8 to 20.8)	

Analysis of covariance was used to test statistical significance of differences in mean change between groups that met (n = 7) or did not meet (n = 42) WHO’s resistance training exercise recommendation. Difference in mean change was adjusted for age, BMI, and baseline value of the outcome. Abbreviations: World Health Organization (WHO), Functional Assessment of Cancer Therapy-General (FACT-G), well-being (WB), FACT-Breast (FACT-B), Trial Outcome Index (TOI), breast cancer (BC), endocrine symptoms (ES), Perceived Stress Scale (PSS), Center for Epidemiologic Studies-Depression (CES-D), State-Trait Anxiety Inventory (STAI), body mass index (BMI).

## Discussion

Consistent with the reported negative effects of chemotherapy on QoL [6] and exercise capacity [10], self-reported QoL as well as resistance and aerobic exercise (at 12 weeks) declined with treatment. Meeting WHO’s aerobic or resistance training exercise recommendation was not associated with a better QoL. DHA supplementation during NAC did not reduce the declines in QoL, aerobic exercise volume or resistance training frequency. A recent systematic review examined RCTs and experimental studies that investigated the effects of supplementation or consumption of foods enriched in n-3 fatty acids (300–6000 mg/day) in breast cancer patients that were receiving treatment or were in the follow-up period [34]. The authors concluded that n-3 fatty acid supplementation led to a significant decrease in perceived stress, sleep disturbance, depression, pain, joint stiffness and fatigue. Among the studies reviewed, only one considered QoL among breast cancer patients that were supplemented with n-3 fatty acids *during* NAC [35]. It consisted of an RCT in which the treatment group received 2.4 g/day of n-3 PUFAs (1.6 g eicosapentaenoic acid (EPA) and 0.8 g DHA) during the six months of chemotherapy. Both groups experienced an increase at three and six months in fatigue, nausea, drowsiness, appetite and dyspnea and there were no significant differences between groups [35]. These findings are consistent with the current study, which reported an increase in fatigue from baseline to the end of chemotherapy, and no difference between the DHA and placebo groups.

An earlier review done by Newell *et al*. (2021) concluded that supplementation of EPA and DHA during cancer therapy improved overall QoL among patients with various types of cancer [14]. Focusing on breast cancer patients, Martinez *et al*. (2019) carried out a single arm clinical trial in which patients were supplemented with n-3 fatty acids (1.4 g EPA + DHA), hydroxytyrosol and curcumin for one month during hormonal therapy [36]. The authors observed a 21.5% decrease in patients’ pain score obtained from the brief pain inventory (BPI) after 30 days of treatment. However, Shen *et al*. (2018) found in an RCT with women undergoing hormonal therapy for breast cancer, supplementation of 3.3 g/day of EPA + DHA for 24 weeks significantly decreased the BPI worst pain scores among obese patients, but there were no differences in treatment arms among non-obese patients [37]. Another RCT supplemented breast cancer patients with 0.2 g EPA and 1.0 g DHA per day for 16 weeks during treatment and one month following treatment, and the authors observed a significant reduction in peripheral neuropathy in the treatment group compared to the control group [38]. The dose, different combinations of EPA and DHA, duration of supplementation, different assessment tools, cancer types and treatments may contribute to the different findings regarding the effects of DHA supplementation on QoL. For example, the breast cancer studies reviewed by Newell *et al*. (2021) consist of participants receiving hormonal therapy, which may affect patients’ QoL differently than chemotherapy [39].

Aerobic exercise volume and resistance training frequency declined over time in the DHA WIN cohort. Mean aerobic exercise volume was significantly lower at weeks 12 and 18 compared to baseline, while mean resistance training frequency was significantly lower at week 12 compared to baseline. This was expected as other studies have reported a decrease in physical activity following a breast cancer diagnosis and during treatment [40–42]. Nielsen *et al*. (2020) performed a qualitative evaluation that looked at barriers to physical activity during chemotherapy and identified recurring themes from patient interviews that contributed to this decline in physical activity, including side effects of chemotherapy as well as a need for education about physical activity during treatment [43,44]. Confusion and a perceived lack of guidance from their oncologist were common reasons stated for not performing physical activity during treatment [43]. The results from the current study suggest that there may be critical points during treatment (i.e., weeks 12 and 18) that require additional supportive care to help prevent the decline in patients’ physical activity.

The American College of Sports Medicine (ACSM) produced a roundtable report in 2018 that detailed the type and duration of exercise shown to improve specific cancer-related side effects [45]. They concluded that moderate-intensity aerobic training for a minimum of 30 minutes at least three times per week, for a minimum of 8–12 weeks positively effects health-related outcomes including anxiety, depression, fatigue, QoL and physical function. They also found that resistance training at least twice weekly in addition to the aerobic exercise recommendation resulted in similar benefits to aerobic exercise alone. Overall, the ACSM recommends avoiding inactivity and attaining the current physical activity guidelines for health (150 minutes/week of aerobic exercise and 2 times/week of strength training) [46]. Unlike the conclusions made by ACSM, patients that reported meeting WHO’s aerobic (≥150 minutes/week) or resistance training (≥2 times/week) exercise recommendation did not have improved anxiety, depression or fatigue. These differences may be due, in part, to the fact that few studies that informed the ACSM guidelines were in the neoadjuvant setting [45], which may result in different side effects than the adjuvant setting [6]. Further, only 29% of participants met WHO’s aerobic exercise recommendation, and 14% met the resistance training recommendation, resulting in small group sizes upon stratification. This proportion is similar to the study discussed by Nielsen *et al*. (2019), in which only 23.3% of the sample reported achieving the recommendation of 150 minutes of moderate to vigorously intense physical activity per week during chemotherapy. The low percentages of cancer survivors meeting these guidelines highlights the need for increased awareness and education, referrals to counseling for physical activity and additional individual and community programs to help patients achieve the recommended levels [47].

There are several strengths with the current study. The DHA WIN clinical trial was the first phase II RCT to supplement DHA during neoadjuvant chemotherapy in women with non-metastatic breast cancer [17]. The DHA and self-administered exercise and QoL questionnaires were cost-effective and resulted in a small respondent burden. Self-administered exercise (Godin Leisure-Time) and QoL (FACT and FACIT) questionnaires used have a low-respondent burden, are cost-effective and have been validated for use in cancer patients [20,33]. The study was also subject to limitations. The DHA WIN RCT was powered to assess changes in the Ki67 index in the tumour [17] and not exercise or QoL. Lack of statistical significance observed for QoL and exercise outcomes may be due, in part, to lack of power to evaluate these secondary outcomes. Therefore, the results can only be interpreted as exploratory and require a larger sample size to make definitive conclusions. For example, the estimated minimal clinically important difference is 4 points for the FACT-General scale [48]. A group sample size of 194 patients would be required to achieve 80% power to detect a difference of 4 points between treatment groups for this scale. Recall and response bias are inherent with self-administered questionnaires [49]. More quantitative techniques should be used to measure exercise in future trials, such as accelerometers, wearable technology and more in-depth questionnaires, as described for other patient populations [50].

In summary, the current exploratory study did not find that DHA supplementation during NAC prevented a decline in QoL or exercise behaviour during treatment. Aerobic and resistance training exercise levels decreased below baseline at various timepoints throughout the trial. Meeting WHO’s aerobic and resistance training exercise guidelines was only achieved by 29% and 14% of the patients, respectively, and was not associated with better QoL. Generally, QoL and exercise decreased throughout the trial, highlighting the need for supportive care and potential therapies that may mitigate these changes in patients receiving cancer treatment. Adequately powered studies are needed to determine if DHA supplementation improves these two indices of health.

## Supporting information

S1 File Baseline Quality of Life Questionnaire. (PDF)

S2 File Post-intervention Quality of Life Questionnaire. (PDF)

S3 File DHA-WIN Study Protocol. (DOCX)

S4 File Consort Checklist. (DOC)

## References

[1] World Health Organization. Global cancer burden growing, amidst mounting need for services. 2024 [cited 2024 June 30]. Available from: https://www.who.int/news/item/01-02-2024-global-cancer-burden-growing--amidst-mounting-need-for-services. PMC11115397 38438207

[2] Teshome M, Hunt KK. Neoadjuvant therapy in the treatment of breast cancer. Surg Oncol Clin N Am. 2014;23(3):505–23. doi: 10.1016/j.soc.2014.03.006 24882348 PMC4044615

[3] Ortmann O, Blohmer J-U, Sibert NT, Brucker S, Janni W, Wöckel A, et al. Current clinical practice and outcome of neoadjuvant chemotherapy for early breast cancer: analysis of individual data from 94,638 patients treated in 55 breast cancer centers. J Cancer Res Clin Oncol. 2023;149(3):1195–209. doi: 10.1007/s00432-022-03938-x 35380257 PMC9984341

[4] Burguin A, Diorio C, Durocher F. Breast cancer treatments: updates and new challenges. J Pers Med. 2021;11(8):808. doi: 10.3390/jpm11080808 34442452 PMC8399130

[5] Sitlinger A, Zafar SY. Health-related quality of life: the impact on morbidity and mortality. Surg Oncol Clin N Am. 2018;27(4):675–84. doi: 10.1016/j.soc.2018.05.008 30213412 PMC6428416

[6] Zhao Y, Chen L, Zheng X, Shi Y. Quality of life in patients with breast cancer with neoadjuvant chemotherapy: a systematic review. BMJ Open. 2022;12(11):e061967. doi: 10.1136/bmjopen-2022-061967 36400735 PMC9677026

[7] Takada K, Kashiwagi S, Fukui Y, Goto W, Asano Y, Morisaki T, et al. Prognostic value of quality-of-life scores in patients with breast cancer undergoing preoperative chemotherapy. BJS Open. 2018;3(1):38–47. doi: 10.1002/bjs5.50108 30734014 PMC6354182

[8] Lee S, Jung S, Jung S, Moon JY, Oh GH, Yeom C-W, et al. Psychiatric symptoms mediate the effect of resilience on health-related quality of life in patients with breast cancer: longitudinal examination. Psychooncol. 2022;31(3):470–7. doi: 10.1002/pon.5829 34668264

[9] Misiąg W, Piszczyk A, Szymańska-Chabowska A, Chabowski M. Physical activity and cancer care-a review. Cancers (Basel). 2022;14(17):4154. doi: 10.3390/cancers14174154 36077690 PMC9454950

[10] Aune D, Markozannes G, Abar L, Balducci K, Cariolou M, Nanu N, et al. Physical activity and health-related quality of life in women with breast cancer: a meta-analysis. JNCI Cancer Spectr. 2022;6(6):pkac072. doi: 10.1093/jncics/pkac072 36474321 PMC9727071

[11] Chen X, Li J, Chen C, Zhang Y, Zhang S, Zhang Y, et al. Effects of exercise interventions on cancer-related fatigue and quality of life among cancer patients: a meta-analysis. BMC Nurs. 2023;22(1):200. doi: 10.1186/s12912-023-01363-0 37312185 PMC10261838

[12] Kawahara T, Iwamoto T, Takashima I, Hanazawa R, Uemura K, Uemura Y, et al. Association of change in health-related quality of life and treatment discontinuation in metastatic breast cancer: a post hoc, exploratory analysis of two randomized clinical trials. Support Care Cancer. 2022;30(10):8367–75. doi: 10.1007/s00520-022-07283-0 35857127 PMC9512887

[13] Morland SL, Martins KJB, Mazurak VC. n-3 polyunsaturated fatty acid supplementation during cancer chemotherapy. J Nutr Interm Metab. 2016; 5:107-16. doi: 10.1016/j.jnim.2016.05.001

[14] Newell M, Mazurak V, Postovit LM, Field CJ. N-3 long-chain polyunsaturated fatty acids, eicosapentaenoic and docosahexaenoic acid, and the role of supplementation during cancer treatment: a scoping review of current clinical evidence. Cancers (Basel). 2021;13(6):1206. doi: 10.3390/cancers13061206 33801979 PMC8000768

[15] Newell M, Goruk S, Schueler J, Mazurak V, Postovit L-M, Field CJ. Docosahexaenoic acid enrichment of tumor phospholipid membranes increases tumor necroptosis in mice bearing triple negative breast cancer patient-derived xenografts. J Nutr Biochem. 2022;107:109018. doi: 10.1016/j.jnutbio.2022.109018 35489658

[16] World Health Organization. WHO guidelines on physical acitvity and sedentary behaviour. 2020 [cited 2024 July 12]. Available from: https://www.who.int/publications/i/item/9789240015128. 33369898

[17] Newell M, Mackey JR, Bigras G, Alvarez-Camacho M, Goruk S, Ghosh S, et al. Comparing docosahexaenoic acid (DHA) concomitant with neoadjuvant chemotherapy versus neoadjuvant chemotherapy alone in the treatment of breast cancer (DHA WIN): protocol of a double-blind, phase II, randomised controlled trial. BMJ Open. 2019;9(9):e030502. doi: 10.1136/bmjopen-2019-030502 31530611 PMC6756327

[18] Cella DF, Tulsky DS, Gray G, Sarafian B, Linn E, Bonomi A, et al. The functional assessment of cancer therapy scale: development and validation of the general measure. J Clin Oncol. 1993;11(3):570–9. doi: 10.1200/JCO.1993.11.3.570 8445433

[19] FACIT.org. Functional assessment of cancer therapy - General. 2024 [cited 2024 February 25]. Available from: https://www.facit.org/measures/fact-g.

[20] Brady MJ, Cella DF, Mo F, Bonomi AE, Tulsky DS, Lloyd SR, et al. Reliability and validity of the functional assessment of cancer therapy-breast quality-of-life instrument. J Clin Oncol. 1997;15(3):974–86. doi: 10.1200/JCO.1997.15.3.974 9060536

[21] FACIT.org. Functional assessment of cancer therapy - Breast. 2024 [cited 2024 February 26]. Available from: https://www.facit.org/measures/fact-b.

[22] FACIT.org. Functional assessment of cancer therapy - Taxane. 2024 [cited 2024 February 26]. Available from: https://www.facit.org/measures/fact-taxane.

[23] Cella D, Peterman A, Hudgens S, Webster K, Socinski MA. Measuring the side effects of taxane therapy in oncology: the functional assesment of cancer therapy-taxane (FACT-taxane). Cancer. 2003;98(4):822–31. doi: 10.1002/cncr.11578 12910528

[24] FACT.org. Functional assessment of cancer therapy - Endocrine symptoms. 2024 [cited 2024 February 26]. Available from: https://www.facit.org/measures/fact-es.

[25] Fallowfield LJ, Leaity SK, Howell A, Benson S, Cella D. Assessment of quality of life in women undergoing hormonal therapy for breast cancer: validation of an endocrine symptom subscale for the FACT-B. Breast Cancer Res Treat. 1999;55(2):189–99. doi: 10.1023/a:1006263818115 10481946

[26] FACIT.org. Functional assessment of chronic illness therapy - Fatigue. 2024 [cited 2024 February 25]. Available from: https://www.facit.org/measures/facit-fatigue.

[27] Yellen SB, Cella DF, Webster K, Blendowski C, Kaplan E. Measuring fatigue and other anemia-related symptoms with the Functional Assessment of Cancer Therapy (FACT) measurement system. J Pain Symptom Manage. 1997;13(2):63–74. doi: 10.1016/s0885-3924(96)00274-6 9095563

[28] Cohen S, Kamarck T, Mermelstein R. A global measure of perceived stress. J Health Soc Behav. 1983;24(4): 385-–96. doi: 10.2307/2136404 6668417

[29] Fordyce MW. A review of research on the happiness measures: a sixty second index of happiness and mental health. Soc Indic Res. 1988;20(4):355–81. doi: 10.1007/bf00302333

[30] Radloff LS. The CES-D scale. Appl Psych Meas. 1977;1(3):385–401. doi: 10.1177/014662167700100306

[31] Skapinakis P. Spielberger state-trait anxiety inventory. Encyclopedia Qual Life Well-Being Res. 2014:6261–4. doi: 10.1007/978-94-007-0753-5_2825

[32] Godin G, Shephard RJ. A simple method to assess exercise behavior in the community. Can J Appl Sport Sci. 1985;10(3):141–6. 4053261

[33] Amireault S, Godin G, Lacombe J, Sabiston CM. Validation of the godin-shephard leisure-time physical activity questionnaire classification coding system using accelerometer assessment among breast cancer survivors. J Cancer Surviv. 2015;9(3):532–40. doi: 10.1007/s11764-015-0430-6 25666749

[34] Osouli-Tabrizi S, Mehdizadeh A, Naghdi M, Sanaat Z, Vahed N, Farshbaf-Khalili A. The effectiveness of omega-3 fatty acids on health outcomes in women with breast cancer: a systematic review. Food Sci Nutr. 2023;11(8):4355–71. doi: 10.1002/fsn3.3409 37576056 PMC10420771

[35] de la Rosa Oliva F, Meneses García A, Ruiz Calzada H, Astudillo de la Vega H, Bargalló Rocha E, Lara-Medina F, et al. Effects of omega-3 fatty acids supplementation on neoadjuvant chemotherapy-induced toxicity in patients with locally advanced breast cancer: a randomized, controlled, double-blinded clinical trial. Nutr Hosp. 2019;36(4):769–76. doi: 10.20960/nh.2338 31192682

[36] Martínez N, Herrera M, Frías L, Provencio M, Pérez-Carrión R, Díaz V, et al. A combination of hydroxytyrosol, omega-3 fatty acids and curcumin improves pain and inflammation among early stage breast cancer patients receiving adjuvant hormonal therapy: results of a pilot study. Clin Transl Oncol. 2019;21(4):489–98. doi: 10.1007/s12094-018-1950-0 30293230

[37] Shen S, Unger JM, Crew KD, Till C, Greenlee H, Gralow J, et al. Omega-3 fatty acid use for obese breast cancer patients with aromatase inhibitor-related arthralgia (SWOG S0927). Breast Cancer Res Treat. 2018;172(3):603–10. doi: 10.1007/s10549-018-4946-0 30159789 PMC6681817

[38] Ghoreishi Z, Esfahani A, Djazayeri A, Djalali M, Golestan B, Ayromlou H, et al. Omega-3 fatty acids are protective against paclitaxel-induced peripheral neuropathy: a randomized double-blind placebo controlled trial. BMC Cancer. 2012;12:355. doi: 10.1186/1471-2407-12-355 22894640 PMC3459710

[39] Ferreira AR, Di Meglio A, Pistilli B, Gbenou AS, El-Mouhebb M, Dauchy S, et al. Differential impact of endocrine therapy and chemotherapy on quality of life of breast cancer survivors: a prospective patient-reported outcomes analysis. Ann Oncol. 2019;30(11):1784–95. doi: 10.1093/annonc/mdz298 31591636

[40] Littman AJ, Tang M-T, Rossing MA. Longitudinal study of recreational physical activity in breast cancer survivors. J Cancer Surviv. 2010;4(2):119–27. doi: 10.1007/s11764-009-0113-2 20180037

[41] Huy C, Schmidt ME, Vrieling A, Chang-Claude J, Steindorf K. Physical activity in a German breast cancer patient cohort: one-year trends and characteristics associated with change in activity level. Eur J Cancer. 2012;48(3):297–304. doi: 10.1016/j.ejca.2011.08.005 21920730

[42] Courneya KS, Friedenreich CM. Relationship between exercise during treatment and current quality of life among survivors of breast cancer. J Psych Oncol. 1997;15(3–4):35–57. doi: 10.1300/j077v15n03_02

[43] Nielsen AM, Welch WA, Gavin KL, Cottrell AM, Solk P, Torre EA, et al. Preferences for mHealth physical activity interventions during chemotherapy for breast cancer: a qualitative evaluation. Support Care Cancer. 2020;28(4):1919–28. doi: 10.1007/s00520-019-05002-w 31367917 PMC6992480

[44] Courneya KS, McKenzie DC, Reid RD, Mackey JR, Gelmon K, Friedenreich CM, et al. Barriers to supervised exercise training in a randomized controlled trial of breast cancer patients receiving chemotherapy. Ann Behav Med. 2008;35(1):116–22. doi: 10.1007/s12160-007-9009-4 18347912

[45] Campbell KL, Winters-Stone KM, Wiskemann J, May AM, Schwartz AL, Courneya KS, et al. Exercise guidelines for cancer survivors: consensus statement from international multidisciplinary roundtable. Med Sci Sports Exerc. 2019;51(11):2375–90. doi: 10.1249/MSS.0000000000002116 31626055 PMC8576825

[46] American College of Sports Medicine. Effects of exercise on health-related outcomes in those with cancer. 2022 [cited 2024 July 12]. Available from: https://www.acsm.org/docs/default-source/files-for-resource-library/cancer-infographic-sept-2022.pdf.

[47] Kenfield SA, Chan JM. Meeting exercise recommendations is beneficial for cancer survivors. J Clin Oncol. 2023;41(32):4965–7. doi: 10.1200/JCO.23.01528 37729601

[48] Cella D, Eton DT, Lai J-S, Peterman AH, Merkel DE. Combining anchor and distribution-based methods to derive minimal clinically important differences on the Functional Assessment of Cancer Therapy (FACT) anemia and fatigue scales. J Pain Symptom Manage. 2002;24(6):547–61. doi: 10.1016/s0885-3924(02)00529-8 12551804

[49] Prince SA, Adamo KB, Hamel ME, Hardt J, Connor Gorber S, Tremblay M. A comparison of direct versus self-report measures for assessing physical activity in adults: a systematic review. Int J Behav Nutr Phys Act. 2008;5:56. doi: 10.1186/1479-5868-5-56 18990237 PMC2588639

[50] Ainsworth B, Cahalin L, Buman M, Ross R. The current state of physical activity assessment tools. Prog Cardiovasc Dis. 2015;57(4):387–95. doi: 10.1016/j.pcad.2014.10.005 25446555

