# Supplementary material for: Exploratory outcomes of the DHA WIN randomized controlled trial: Supplementing women with docosahexaenoic acid did not reduce the impact of neoadjuvant breast cancer chemotherapy on quality of life or exercise behaviour
Source: PLoS One. 2025 May 2;20(5):e0322178. doi: 10.1371/journal.pone.0322178 (PMC12047813; doi:10.1371/journal.pone.0322178)
Supplement: S2 File — (PDF) [file pone.0322178.s002.pdf]

Participant No: _______________ Date completed: ___________________

Post-intervention Quality of Life Questionnaire

Docosahexaenoic acid (DHA) for Women with Breast Cancer in the Neoadjuvant Setting (DHA WIN)

Post-intervention Questionnaire

Instructions

Thank you for your continued participation in this study. At this post-intervention assessment, we are going to ask you many of the same questions as in the previous questionnaires. However, it is important to answer these questions based on what you are thinking and feeling right now, and not on how you answered the questions the last time. This will give us important information about how your thoughts and feelings have changed. Many of the questions may seem similar but it is important to treat each question separately and provide an answer for each. Also, if at all possible, it is important to answer all questions. However, if you feel uncomfortable answering certain questions please leave them blank. All responses are completely confidential and will never be used in any way that could link them to you. There are no right or wrong answers and all we ask is that you provide responses that are as honest and accurate as possible. The questionnaire should take about 30-45 minutes of your time to complete.

Below is a list of statements that other people with cancer have said are important to their quality of life. Please indicate the extent to which you have experienced each of the statements during the past 7 days by circling the appropriate number using the following scale.

During the PAST WEEK:	not at all	a little bit	some-what	quite a bit	very much
1. I have a lack of energy	0	1	2	3	4
2. I have nausea	0	1	2	3	4
3. Because of my physical condition, I have trouble meeting the needs of my family	0	1	2	3	4
4. I have pain	0	1	2	3	4
5. I am bothered by side effects of treatment	0	1	2	3	4
6. I feel sick	0	1	2	3	4
7. I am forced to spend time in bed	0	1	2	3	4
8. I feel close to my friends	0	1	2	3	4
9. I get emotional support from my family	0	1	2	3	4
10. I get support from my friends	0	1	2	3	4
11. My family has accepted my illness	0	1	2	3	4
12. I am satisfied with family communication about my illness	0	1	2	3	4
13. I feel close to my partner (or the person who is my main support)	0	1	2	3	4
14. I am satisfied with my sex life	0	1	2	3	4
15. I feel sad	0	1	2	3	4
16. I am satisfied with how I am coping with my illness	0	1	2	3	4

During the <u>PAST WEEK</u>:

	not at all	a little bit	some-what	quite a bit	very much
17. I am losing hope in the fight against my illness	0	1	2	3	4
18. I feel nervous	0	1	2	3	4
19. I worry about dying	0	1	2	3	4
20. I worry that my condition will get worse	0	1	2	3	4
21. I am able to work (include work at home)	0	1	2	3	4
22. My work (include work at home) is fulfilling	0	1	2	3	4
23. I am able to enjoy life	0	1	2	3	4
24. I have accepted my illness	0	1	2	3	4
25. I am sleeping well	0	1	2	3	4
26. I am enjoying the things I usually do for fun	0	1	2	3	4
27. I am content with the quality of my life right now	0	1	2	3	4
28. I have been short of breath	0	1	2	3	4
29. I am self-conscious about the way I dress	0	1	2	3	4
30. My arms are swollen or tender	0	1	2	3	4
31. I feel sexually attractive	0	1	2	3	4
32. I have been bothered by hair loss	0	1	2	3	4
33. I worry about the risk of cancer in my family	0	1	2	3	4
34. I worry about the effect of stress on my illness	0	1	2	3	4
35. I am bothered by a change in weight	0	1	2	3	4
36. I am able to feel like a woman	0	1	2	3	4
37. I have certain parts of my body where I experience significant pain.	0	1	2	3	4

During the PAST WEEK:

FATIGUE SYMPTOMS

	not at all	a little bit	some-what	quite a bit	very much
1. I feel fatigued	0	1	2	3	4
2. I feel weak all over	0	1	2	3	4
3. I feel listless ("washed out")	0	1	2	3	4
4. I feel tired	0	1	2	3	4
5. I have trouble starting things because I am tired	0	1	2	3	4
6. I have trouble finishing things because I am tired	0	1	2	3	4
7. I have energy	0	1	2	3	4
8. I am able to do my usual activities	0	1	2	3	4
9. I need to sleep during the day	0	1	2	3	4
10. I am too tired to eat	0	1	2	3	4
11. I need help doing my usual activities	0	1	2	3	4
12. I am frustrated by being too tired to do the things I want to do	0	1	2	3	4
13. I have to limit my social activity because I am tired	0	1	2	3	4

During the PAST WEEK:

TAXANE SYMPTOMS

	not at all	a little bit	some-what	quite a bit	very much
1. I have numbness or tingling in my hands	0	1	2	3	4
2. I have numbness or tingling in my feet	0	1	2	3	4
3. I feel discomfort in my hands	0	1	2	3	4
4. I feel discomfort in my feet	0	1	2	3	4
5. I have joint pain or muscle cramps	0	1	2	3	4
6. I feel weak all over	0	1	2	3	4
7. I have trouble hearing	0	1	2	3	4
8. I get a ringing or buzzing in my ears	0	1	2	3	4
9. I have trouble buttoning buttons	0	1	2	3	4
10. I have trouble feeling the shape of small objects when they are in my hand	0	1	2	3	4
11. I have trouble walking	0	1	2	3	4
12. I feel bloated	0	1	2	3	4
13. My hands are swollen	0	1	2	3	4
14. My legs or feet are swollen	0	1	2	3	4
15. I have pain in my fingertips	0	1	2	3	4
16. I am bothered by the way my hands or nails look	0	1	2	3	4

During the PAST WEEK:

ENDOCRINE SYMPTOMS

	not at all	a little bit	some-what	quite a bit	very much
1. I have hot flashes	0	1	2	3	4
2. I have cold sweats	0	1	2	3	4
3. I have night sweats	0	1	2	3	4
4. I have vaginal discharge	0	1	2	3	4
5. I have vaginal itching/irritation	0	1	2	3	4
6. I have vaginal bleeding or spotting	0	1	2	3	4
7. I have vaginal dryness	0	1	2	3	4
8. I have pain or discomfort with intercourse	0	1	2	3	4
9. I have lost interest in sex	0	1	2	3	4
10. I have gained weight	0	1	2	3	4
11. I feel lightheaded (dizzy)	0	1	2	3	4
12. I have been vomiting	0	1	2	3	4
13. I have diarrhea	0	1	2	3	4
14. I get headaches	0	1	2	3	4
15. I feel bloated	0	1	2	3	4
16. I have breast sensitivity/tenderness	0	1	2	3	4
17. I have mood swings	0	1	2	3	4
18. I am irritable	0	1	2	3	4
19. I have pain in my joints	0	1	2	3	4

The questions in this scale ask you about your feelings and thoughts during the last month. Although some of the questions are similar, there are differences between them and you should treat each one as a separate question. The best approach is to answer each one fairly quickly. For each question, please choose from the following alternatives:

	0	1	2	3	4
	never	almost never	some-times	fairly often	very often
**In the last month, how often have you…**					
1. been upset because of something that happened unexpectedly	0	1	2	3	4
2. felt that you were unable to control the important things in your life	0	1	2	3	4
3. felt nervous and stressed	0	1	2	3	4
4. dealt successfully with irritating life hassles	0	1	2	3	4
5. felt that you were effectively coping with important changes that were occurring in your life	0	1	2	3	4
6. felt confident about your ability to handle your personal problems	0	1	2	3	4
7. felt that things were going your way	0	1	2	3	4
8. found that you could not cope with all the things that you had to do	0	1	2	3	4
9. been able to control irritations in your life	0	1	2	3	4
10. felt that you were on top of things	0	1	2	3	4
11. been angered because of things that happened that were outside of your control	0	1	2	3	4
12. found yourself thinking about things that you have to accomplish	0	1	2	3	4
13. been able to control the way you spend your time	0	1	2	3	4
14. felt difficulties were piling up so high that you could not overcome them	0	1	2	3	4

The following question asks you to rate, on average, how happy or unhappy you felt over the past week. Please read all the statements first and then check the one statement (between 0 and 10) that best describes your average level of happiness over the past week. Check only ONE item.

On average, over the PAST WEEK I have felt:

_____ 10. Extremely happy (feeling ecstatic, joyous, fantastic!).

_____ 9. Very happy (feeling really good, elated!).

_____ 8. Pretty happy (spirits high, feeling good).

_____ 7. Mildly happy (feeling fairly good, somewhat cheerful).

_____ 6. Slightly happy (just a bit above neutral).

_____ 5. Neutral (not particularly happy or unhappy).

_____ 4. Slightly unhappy (just a bit below neutral).

_____ 3. Mildly unhappy (just a little low).

_____ 2. Pretty unhappy (somewhat "blue," spirits down).

_____ 1. Very unhappy (depressed, spirits very low).

_____ 0. Extremely unhappy (utterly depressed, completely down).

This next question asks you to estimate the percentage of time, on average, that you felt happy, unhappy, and neutral (neither happy nor unhappy) over the past week. Write down your best estimates in the spaces below. Make sure the three figures add up to 100 percent.

Over the PAST WEEK:

The percentage of time I felt happy was: _______%

The percentage of time I felt unhappy was: _______%

The percentage of time I felt neutral was: _______%

Total: 100 %

Below is a list of statements concerning how you might have felt or behaved in the past week. Please use the following scale to indicate how often you felt or behaved in these ways in the past week.

0	1	2	3
Rarely or none of the time (< 1 day)	Some of the time (1-2 days)	Much of the time (3-4 days)	Most or all of the time (5-7 days)

During the PAST WEEK:

1. I felt depressed.	0	1	2	3
2. I felt that everything I did was an effort.	0	1	2	3
3. My sleep was restless.	0	1	2	3
4. I was happy.	0	1	2	3
5. I felt lonely.	0	1	2	3
6.People were unfriendly.	0	1	2	3
7. I enjoyed life.	0	1	2	3
8. I felt sad.	0	1	2	3
9. I felt that people disliked me.	0	1	2	3
10. I could not get "going".	0	1	2	3

A number of statements which people have used to describe themselves are given below. Read each statement and then circle the appropriate number that best indicates how you have felt during the past week. There are no right or wrong answers. Do not spend too much time on any one statement but give the answer that best describes how you felt.

During the PAST WEEK:

	not at all	somewhat	moderately so	very much so
1. I felt calm	1	2	3	4
2. I was tense	1	2	3	4
3. I felt at ease	1	2	3	4
4. I worried over possible misfortunes	1	2	3	4
5. I felt frightened	1	2	3	4
6. I felt self-confident	1	2	3	4
7. I was jittery	1	2	3	4
8. I was relaxed	1	2	3	4
9. I was worried	1	2	3	4
10. I felt steady	1	2	3	4
