# Supplementary material for: Exploratory outcomes of the DHA WIN randomized controlled trial: Supplementing women with docosahexaenoic acid did not reduce the impact of neoadjuvant breast cancer chemotherapy on quality of life or exercise behaviour
Source: PLoS One. 2025 May 2;20(5):e0322178. doi: 10.1371/journal.pone.0322178 (PMC12047813; doi:10.1371/journal.pone.0322178)
Supplement: S3 File — (DOCX) [file pone.0322178.s003.docx]

**Docosahexaenoic acid (DHA) for Women with Breast Cancer in the Neoadjuvant Setting**

Protocol number: IIT-0005

Version date 1.4: April 7 2021

Clinical Trial Registration: NCT03831178

# Study Summary/ Synopsis

| Title | **DHA** for **W**omen with Breast Cancer **I**n the **N**eoadjuvant Setting |
| --- | --- |
| Short Title | DHA WIN |
| Protocol Number | IIT-005 |
| Phase | Phase IIB |
| Methodology | Double blind randomized study to examine if DHA supplementation alters efficacy of chemotherapy, using change in Ki67 labelling index as a surrogate marker of efficacy. |
| Study Duration | 2.5 years |
| Study Center | Single-center (Cross Cancer Institute) |

| Objectives | Primary Objective:  1. Determine the efficacy of supplemental DHA provided with standard neoadjuvant chemotherapy in patients with invasive breast cancer as measured by change in Ki67 index from biopsy to surgical excision.  Secondary Objectives:   1. Assess DHA incorporation into plasma phospholipids to establish the dose of DHA required to increase efficacy of chemotherapy in breast cancer patients undergoing neoadjuvant chemotherapy treatment. 2. Examine effects of DHA supplementation on immune function (plasma cytokines, chemokines, and inflammatory markers and systemic immune function) in patients undergoing neoadjuvant chemotherapy. 3. Identify factors that may affect DHA incorporation into plasma phospholipids. 4. Examine changes in markers for apoptosis (Caspase-3) and tumour infiltrating lymphocytes (by immunohistochemistry) following DHA supplementation in patients undergoing neoadjuvant chemotherapy. 5. Describe the rate of pathological complete response in resected breast tissue and axillary nodes in breast cancer patients receiving DHA supplementation in combination with neoadjuvant chemotherapy. 6. Describe the rate of specific chemotherapy associated toxicities. 7. Describe the rate of hospitalization for chemotherapy associated toxicities.   Exploratory Objectives:   1. Assess changes in quality of life following DHA supplementation in patients undergoing neoadjuvant chemotherapy 2. Assess exercise frequency following DHA supplementation in patients undergoing neoadjuvant chemotherapy 3. Assess the rate of breast conservation, specifically the rate of lumpectomy and mastectomy. 4. Analyze the surgical reports to determine if there is a qualitative or quantitative difference between placebo and treatment arms in volume of surgical blood loss. 5. Analyze local control, relapse free survival and overall survival by electronic medical record and / or paper medical chart review at 3, 5, and 10 years to explore possible effects on long- term outcome. |
| --- | --- |
| Number of Subjects | 74: 2 arm trial, 37 participants/arm. Breast cancer patients receiving neoadjuvant chemotherapy prior to surgery |

| Accrual rate and Justification | Breast cancer patients receiving neoadjuvant chemotherapy account for approximately 20% of newly diagnosed breast cancer patients, approximately 10-12/month. Assuming a conservative accrual rate of 30%, accrual is estimated to be completed in 19-24 months with 3-4 patients recruited per month. |
| --- | --- |
| Diagnosis and Main Inclusion Criteria | 1. Chemotherapy naïve women with clinical stage I, II and III breast cancer prescribed neoadjuvant chemotherapy prior to surgery. 2. ECOG Performance status of 0 or 1. 3. Hematology and biochemistry assessments (CBC and differential, PTT, PT/INR, AST, ALP, Bilirubin, and Creatinine) within normal range unless determined not clinically significant by the qualified investigator. 4. Ability to take oral medications. 5. Adequate tissue specimen for diagnosis, biomarkers, and Ki67 analysis. |
| Investigational product, treatment or intervention | DHA: 11 capsules per day containing DHA-enriched triglyceride oil (1 g capsules containing a minimum of 400 mg DHA) for a total of approximately 4.4 g DHA/day, oral, divided into three times daily with meals or as tolerated.  DHA capsules will be produced by DSM Nutritional Products. |
| Duration of administration | Approximately 12-20 weeks (not including delays), Administration will begin on day 1 of the initial cycle of chemotherapy, and continue for 4-6 cycles of chemotherapy up until definitive breast surgery. Administration will end when subject undergoes breast surgery. |
| Reference therapy | Placebo: corn/soy oil blend capsules (11 per day), oral, divided three times daily with meals or as tolerated.  Placebo capsules will be produced by DSM Nutritional Products. |
| Statistical Methodology | Primary End-points: The percent change in Ki67 will be determined as an absolute percentage. The number of patients showing a decrease and the 95% confidence interval for the mean percent change in the Ki67 level from baseline in subjects receiving DHA supplementation will be compared to subjects receiving placebo. Test of proportions will be used to compare the proportions between the two groups. SAS version 9.4 (SAS Institute Inc., Cary, NC) software will be used for statistical analysis. A p-value <0.05 level will be used for all statistical  significance. Two-sided tests will be used for all statistical tests. |

# Table of Contents

[1 Study Summary/ Synopsis 2](#_Toc190158974)

[2 Table of Contents 5](#_Toc190158975)

[3 Introduction 7](#_Toc190158976)

[3.1 Background and Rationale 7](#_Toc190158977)

[3.2 Compliance Statement 14](#_Toc190158978)

[3.3 Description of the population to be studied 14](#_Toc190158979)

[3.4 Trial Objectives and Purpose 15](#_Toc190158980)

[3.5 Trial Design 16](#_Toc190158981)

[3.6 Summary of Trial Endpoints 17](#_Toc190158982)

[3.7 Steps to Minimize Bias 18](#_Toc190158983)

[3.8 Trial Treatment 18](#_Toc190158984)

[3.9 Trial Duration 19](#_Toc190158985)

[3.10 Withdrawal/Discontinuation Criteria 19](#_Toc190158986)

[3.11 Trial Product Accountability 20](#_Toc190158987)

[3.12 Maintenance of Randomization 20](#_Toc190158988)

[3.13 Source Data 21](#_Toc190158989)

[4 Methodology: Participants, Interventions and Outcomes 22](#_Toc190158990)

[4.1 Consent 22](#_Toc190158991)

[4.2 Eligibility Criteria 22](#_Toc190158992)

[4.3 Sample Size 23](#_Toc190158993)

[4.4 Recruitment 24](#_Toc190158994)

[4.5 Study procedures 24](#_Toc190158995)

[4.6 Outcomes, Methodology and Assessment 30](#_Toc190158996)

[5 Methodology: Data Collection, Management, and Analysis 36](#_Toc190158997)

[5.1 Data Collection Methods 36](#_Toc190158998)

[5.2 Statistics 36](#_Toc190158999)

[5.3 Criteria for the Termination of the Trial 38](#_Toc190159000)

[5.4 Deviations 39](#_Toc190159001)

[5.5 Data Management 39](#_Toc190159002)

[6 Quality Control, Quality Assurance and Monitoring 41](#_Toc190159003)

[6.1 Source Data, Documents, and Monitoring 41](#_Toc190159004)

[6.2 Data Monitoring 41](#_Toc190159005)

[6.3 Auditing 43](#_Toc190159006)

[7 Approvals 44](#_Toc190159007)

[7.1 Ethics 44](#_Toc190159008)

[8 Protocol Amendments 44](#_Toc190159009)

[9 Financing and Insurance 45](#_Toc190159010)

[10 Protocol Registration 45](#_Toc190159011)

[11 Publication Policy 45](#_Toc190159012)

[12 References 46](#_Toc190159013)

[13 List of Abbreviations 50](#_Toc190159014)

# Introduction

## Background and Rationale

#### This protocol will evaluate the use of daily supplementation of Docosahexaenoic acid (DHA) in the form of DHA-enriched triglyceride oil in treatment naïve breast cancer patients who will be undergoing neoadjuvant chemotherapy.

Docosahexaenoic acid (DHA) is an omega-3 long chain polyunsaturated fatty acid (n-3 LCPUFA). N-3 LCPUFA are essential fatty acids in the diet. The majority of n-3 LCPUFA in the diet is alpha-linolenic acid (ALA). While DHA can be synthesized from ALA and other n-3 LCPUFA in the body, endogenous synthesis is low ^(1)^. Consequently, the only way to significantly increase levels of DHA in tissues is by directly consuming this fatty acid.

Common sources of DHA are fatty fish, fish oil and omega-3 supplements and fortified foods. DHA is readily incorporated into membrane phospholipids and induces changes in the properties of the cell membrane. These include altered fluidity; permeability and membrane transport as well as affecting the localization and activity of membrane bound receptors and enzymes ^(2; 3)^. It is well established that changes in membrane DHA has pleotropic effects in the body, including modulation of neurological, immune, and cardiovascular functions.

In breast cancer, multiple lines of evidence strongly suggest that DHA has a beneficial effect. DHA increases sensitivity of breast cancer cells to different chemotherapeutic agents ^(4; 5)^, and in animal models of breast cancer, dietary DHA decreases tumour growth^(6; 7)^. Our preclinical studies demonstrate that DHA increases efficacy of both doxorubicin and docetaxel, two agents commonly used in the adjuvant setting for breast cancer treatment. Furthermore, DHA mitigates chemotherapy induced weight loss in mice, and reduces paclitaxel toxicities in breast cancer patients, strongly indicating that DHA protects against toxicity in normal tissues. Directly relevant to this study, increased DHA in breast adipose tissue correlates with improved response to chemotherapy ^(8)^, and increased dietary intake of n-3 LCPUFA, including DHA, results in increased DHA incorporation in breast adipose tissue ^(9)^. Lastly, in advanced metastatic breast cancer, DHA supplementation correlated with improved outcomes in a subset of patients ^(10)^. Consequently, we hypothesize that the therapeutic index (efficacy: toxicity ratio) will be improved with the addition of DHA. In this clinical trial, we will investigate the benefit of DHA supplementation in combination with neoadjuvant chemotherapy in patients with breast cancer.

We propose a pilot study in which women undergoing neoadjuvant chemotherapy treatment will receive DHA supplementation so as to examine if DHA supplementation alters efficacy of chemotherapy, using change in Ki67 labeling index as a surrogate marker of efficacy. We will examine DHA incorporation in a heterogeneous patient population. Based on the pre- clinical and clinical research, DHA incorporation was found to correlate with efficacy of DHA in improving response to chemotherapy in breast cancer. However, DHA incorporation at a given dose appears to be heterogeneous and the source of this heterogeneity is not well characterized such that doses used in future trials may need to be individualized to ensure required level of tissue incorporation for beneficial effect. This study will further investigate the relationship between DHA in plasma phospholipids (as a potential biomarker of tumour incorporation) and effect on systemic immune function.

### Pre-Clinical Studies

Exposure of human breast cancer cell lines to DHA reduces markers of cellular proliferation, and induces apoptosis, as observed by caspase activation, loss of mitochondrial membrane potential, and increased DNA fragmentation ^(11)^. This anti-cancer effect is specific to malignant cells, as cell viability of the non-transformed breast cell line MCF-12A was not affected by similar concentrations of DHA^(4)^. DHA has also been demonstrated to decrease the migration of MDA-MB-231 and other breast cancer cells, and metastasis in a mouse mammary tumour model^(12; 13; 14)^. Part of the mechanism of DHA action may be the elimination breast tumour stem/initiating cells. Treatment of breast cancer cells with DHA decreased cells expressing breast cancer stem cell biomarkers and mammosphere formation^(15; 16)^. Lastly, n-3 LCPUFA are well established as modulators of the immune system ^(17)^. DHA reduces a host of inflammatory responses and can also give rise to bioactive lipids that regulate inflammation including resolvins, protectins, and maresins ^(17; 18)^. In other cancer models, n-3 LCPUFA, including DHA, can reduce inflammation and improve anti-tumour immune function (i.e. T cell and Natural Killer (NK)) (reviewed by ^(19; 20)^.

When combined with systemic treatment, DHA has been demonstrated to potentiate effects of with both cytotoxic chemotherapeutic agents, and to a lesser extent, with trastuzumab and tamoxifen in animal models of breast cancer^(6; 7)^. *In vitro,* DHA increases sensitivity of breast cancer cells to the cytotoxic effects of chemotherapeutic drugs^(21; 22)^. In our lab, we have shown that pre-treatment of breast cancer cell lines with DHA increases the cytotoxicity of doxorubicin in MDA-MB-231 cells^(4)^ (Figure 1). We have validated this in *nu/nu* mice where we fed DHA to mice with implanted MDA-MB-231 tumours ^(23)^. When tumour-bearing mice were fed a diet of DHA 3.8% w/w fat, compared to isocaloric control diet, mice fed the DHA diet had increased DHA in plasma phospholipids compared to mice on control diet with a fatty acid composition more representative of the North American population (4.38± 0.15% DHA vs 1.09±0.42% DHA P<0.05). Mice supplemented with DHA had a significantly reduced tumour burden when treated with doxorubicin compared to mice on control diet (Figure 2). In addition to decreasing tumour burden in combination with doxorubicin, animals fed the DHA diet had reduced weight loss (p<0.05) compared to mice treated with doxorubicin fed the control diet (Figure 3) ^(23)^. Our lab and others have investigated DHA induced chemo-sensitization with additional chemotherapeutics. Docetaxel is a taxane drug commonly used as a first line treatment in breast cancer. When MDA- MB-231 breast cancer cells are incubated with docetaxel in combination with DHA, cell viability is significantly reduced compared to paclitaxel or docetaxel alone ^(5; 24)^. This effect was reproduced in a rodent mammary tumour model ^(5)^. We have just completed studies confirming the beneficial effect of DHA in NOD scid gamma (NSG) mice implanted with breast cancer patient-derived xenografts (PDX). Mice fed a DHA rich diet (3.8%w/w) had significantly smaller tumours than mice fed a control diet, and increased sensitivity to treatment with docetaxel (Figure 4). Analysis of tumour tissue revealed a clear decrease in staining for the proliferation marker Ki67 in tumours from mice fed a DHA rich diet and treated with docetaxel compared to mice on control diet (Figure 5). Furthermore, tumours from mice fed DHA diet had much greater levels of DHA in tumour phospholipids compared to mice fed control diet (4.84±0.22% DHA vs 2.36±0.15% DHA, P<0.05). The PDX used in this study has been found to be resistant to all

cytotoxic drugs and this is the first evidence that the tumour can be sensitized by another compound.


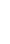

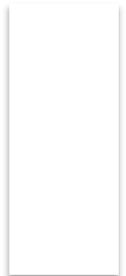

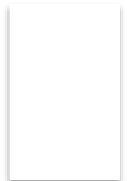

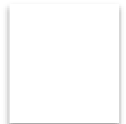

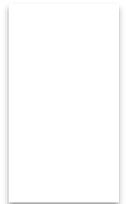

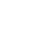

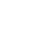

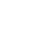

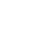

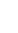

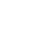

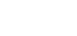

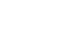

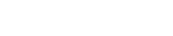

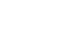

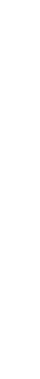

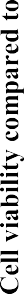

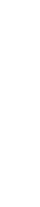

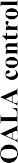

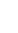

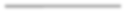

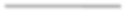

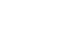

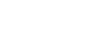

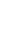

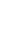

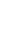

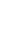

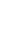

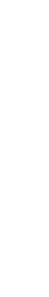

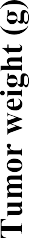

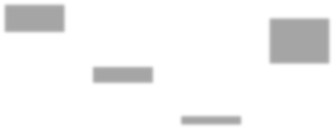

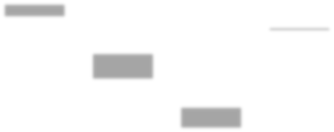

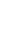

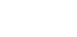

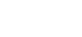

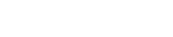

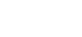

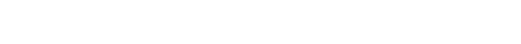

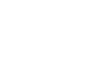

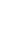

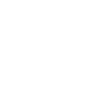

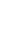

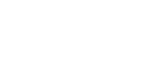

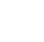

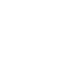

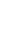


**Diet and Treatment Groups**

**DOX**

**DHA DOX**

**DHA**

**CNT**

2

1

0

c

3

b

4

ab

a

5

**DOX**

**DHA DOX**

**DHA**

**CNT**

0.8

0.6

0.4

0.2

0

0.02

1

0.004

1.2

Figure 1. MDA-MB-231

cells were pretreated with 60 μM DHA or with 40 μM/ 40 μM OALA control medium for 48h followed by treatment with or without DOX (0.22 μg/ml) for 24h. Cellular viability in response to each treatment relative to OALA control was determined by the WST-1 assay. Bars represent the mean ± SEM (n=4) (p<0.05).

Figure 2. Average tumour weight at necropsy. *Nu/nu* mice were injected with MDA-MB-231 cells and maintained on control diet for 4 weeks. 1 week prior to commencing chemotherapy, mice were randomized into control or DHA diet groups and then into (DOX) chemotherapy 5 mg/kg (2x

weekly) or saline control groups for an additional 4 weeks. Values represent the mean ± SEM (n=4). Values that do not share a common letter are significantly different (p<0.05).

Figure 3. Body weights of *nu/nu* mice fed control or DHA diet with or without doxorubicin chemotherapy. Body weights are normalized to 100%. Values represent mean ± SEM (n=4).


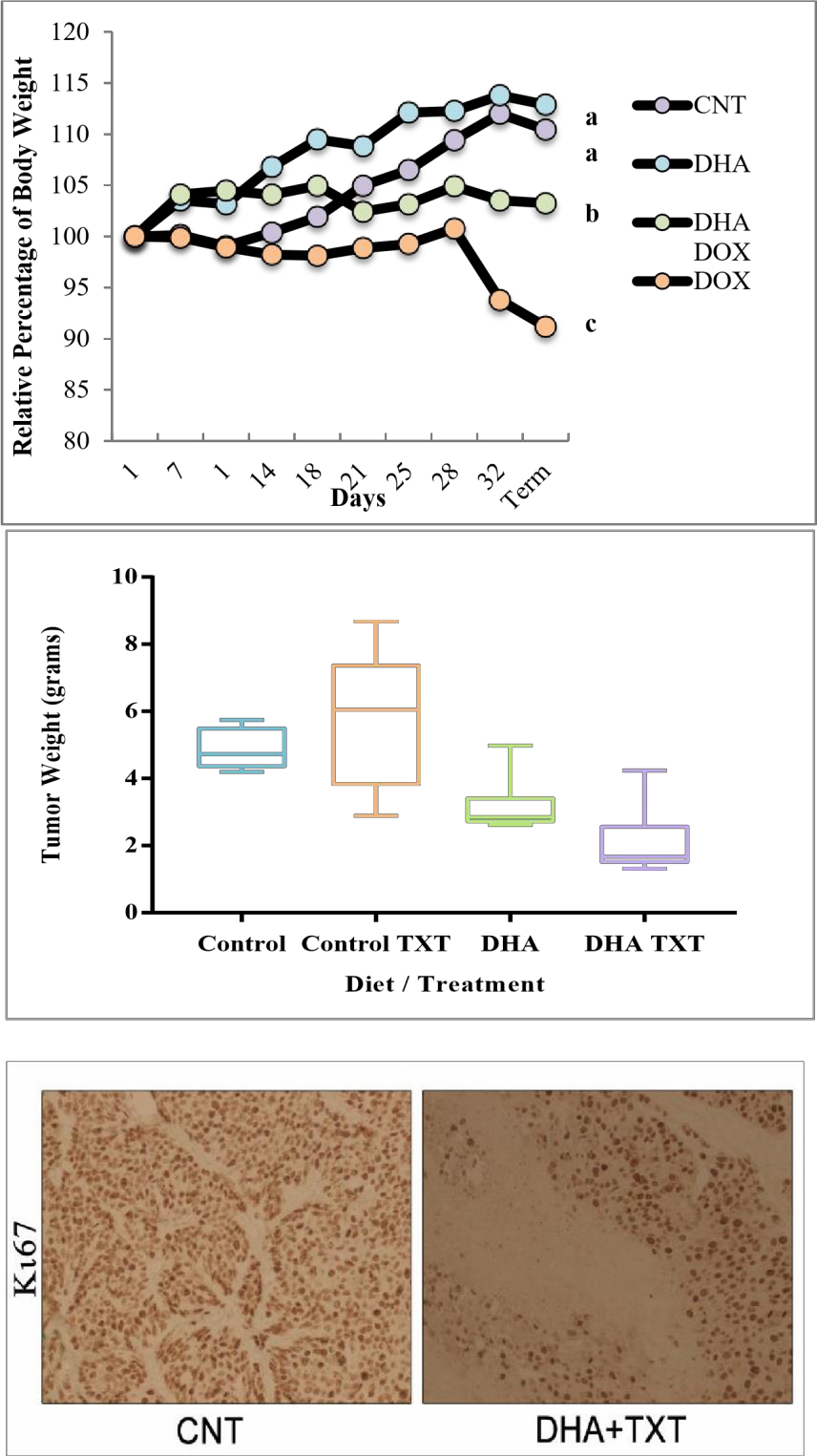


a

a

b

c

Values that do not share a common letter are significantly different (p<0.05).

Figure 4. Average tumour weight at necropsy. Breast cancer patient-derived xenografts were implanted into NOD scid gamma (NSG) mice. Tumour bearing mice (7/group) were randomized to control or DHA rich diet (3.8% w/w DHA) for 1 week, then further randomized to receive IP injections 2X weekly with docetaxel (TXT) (5mg/ kg) or saline control for 6 weeks. Values represent the mean ± SEM (n=7). Values that do not share a common letter are significantly different (p<0.05).

Figure 5. Representative Ki67staining of tumour tissue.

Ki67 staining was detected by immunohistochemistry with antiMIB1 in PDX tumour sections from mice fed control diet (CNT) or DHA diet (3.8% w/w DHA) and treated with 5mg/kg docetaxel for 6 weeks (DHA+TXT).

- - 1. Applicable Clinical Studies

An open label phase II clinical trial provides the only clinical evidence that DHA supplementation may increase response to chemotherapy. In this trial, DHA supplements were given in combination with chemotherapy, beginning seven days prior to beginning chemotherapy, and continued for 5 months while receiving chemotherapy. Chemotherapy consisted of standard first-line treatment regimen FEC 75 (fluorouracil, epirubicin, cyclophosphamide) ^(25)^ on a three-week cycle. Patients with higher incorporation of DHA into plasma phospholipids had significantly increased survival compared to patients with lower levels of DHA incorporation ^(10)^. This trial consisted of twenty-five patients with advanced, metastatic breast cancer who were all given DHA supplements in the form of capsules containing DHA- enriched triglyceride oil (1.8g/day total DHA), taken three times daily with meals^(10)^. This data is suggestive of efficacy but there are several problems that make results difficult to translate. Firstly, the patients had advanced cancers and for the majority of women diagnosed with breast cancer and treated with chemotherapy, survival is not a useful endpoint. Secondly the dose of DHA was very low, compared to what has been used to demonstrate efficacy in pre-clinical animal models. Thirdly, DHA incorporation after a 7- day loading period was highly variable between individuals, ranging from 2.5% to 8.3% of total fatty acids in plasma phospholipids. In patients with high DHA incorporation, overall survival was 34 months, compared to 18 months in patients with low DHA incorporation (P=0.007). This suggests that the dose received by many of the patients may not have been high enough to test efficacy. In support of this, a small prospective study of patients with non-metastatic breast cancer identified DHA as an independent predictive factor of response to chemotherapy. Increased DHA in breast adipose tissue obtained during biopsies correlated with increased response to chemotherapy as determined by clinical and mammographic assessment. This study included 56 patients who were treated with 3 cycles of chemotherapy with mitoxantrone or epirubicin, vindesine, and 5-fluoruracil ^(8)^.

There have been several studies that have examined n-3 LCPUFA supplementation in women at high risk of breast cancer (prevention studies). Supplementation in these studies has ranged from

0.84 g to 7.56 g of n-3 LCPUFA eicosopentaenoic acid (EPA) + DHA ^(9; 26; 27)^. Supplementation induces significant increases in n-3 LCPUFA in plasma phospholipids and in breast adipose tissue. Incorporation of n-3 LCPUFA was observed to be dose-dependent, with higher n-3 LCPUFA supplementation resulting in increased incorporation in plasma and breast adipose phospholipids ^(9)^. Straka et al. ^(27)^ also examined the effect of n-3 LCPUFA source on incorporation. This study found that incorporation of n-3 LCPUFA from dietary fish was similar to incorporation of n-3 LCPUFA obtained from supplementation with n-3 LCPUFA capsules. There was no difference in fatty acid composition of breast adipose tissue or plasma phospholipids between the two sources of n-3 LCPUFA ^(27)^, suggesting that the form of the fatty acid is not important. However, these studies did not examine differences in DHA incorporation between study subjects or the impact of DHA incorporation to effect on breast tissue.

Relative to the current proposal, these dose incorporation studies reported no serious adverse effects or toxicity associated with n-3 LCPUFA supplementation. A small number of participants reported minor side effects related to gastro-intestinal disturbance, and one patient reported easy bruising that was characterized as probably due to supplementation ^(26)^. The use of high doses of n-3 LCPUFA, including DHA, is currently being studied in many pathological inflammatory conditions, including cardiovascular disease and cancer cachexia ^(17; 28; 29)^. Clinical trials examining efficacy of n-3 LCPUFA in treating a number of pathological conditions do not report serious adverse events with high DHA supplementation^(30)^.

In contrast to concerns about adverse effects, DHA may have additional protective against side effects of some chemotherapeutic treatments. A small-randomized control trial examined incidence of peripheral neuropathy in breast cancer patients being treated with paclitaxel and n- 3 LCPUFA supplements. Supplementation with n-3 LCPUFA (345 mg DHA, 65 mg EPA) decreased the incidence of peripheral neuropathy patients^(31)^ (33). Other clinical trials report that supplementation with n-3 LCPUFA at a wide range of doses (0.6g-8.6 g/day) increases tolerability of a number of different chemotherapeutic drugs in a range of malignancies, include lung cancer, pancreatic cancer, and colorectal cancer ^(32)^.

Proliferation in breast tumour biopsies is assessed by immunohistochemical analysis of cells staining positive for the nuclear antigen Ki67^(33)^. The Ki67 nuclear antigen is expressed in all phases of the cell cycle, G_1_, S, G_2_, and M, but not in G_0_ ^(34; 35)^. The intensity of Ki67 staining is frequently used as a primary endpoint to measure efficacy of neoadjuvant therapy in clinical trials. Ki67 change, the change between pre- and posttreatment Ki67, has been reported to be an independent prognostic factor in luminal A, luminal B, triple-negative, and HER2+ breast cancer, and has been reported to be a useful surrogate marker of relapse free survival in luminal B, triple- negative, and HER2+ breast cancer ^(36; 37)^.

Preliminary clinical data from other groups and pre-clinical cell culture and animal models of breast cancer work by our group provide support to move to a clinical trial to determine if supplementation with DHA can increase the efficacy of cytotoxic chemotherapy in breast cancer treatment. When DHA is provided in the diet, there is a significant increase in DHA incorporation into plasma phospholipids, breast adipose tissue, and tumour tissue but the rate and degree to which this occurs may influence efficacy. For example, in advanced metastatic breast cancer patients, DHA incorporation into plasma phospholipids was reported to be heterogeneous, and incorporation positively correlated with overall survival and response to chemotherapy. The proposed exploratory clinical trial will provide indispensable data to move forward into introducing DHA as an adjuvant for breast cancer treatment. This trial is a low-risk pivotal study that can provide information necessary for the practical and efficacious introduction of a safe natural health product to current breast cancer treatment.

- - 1. Name and description of the investigational product, treatment or intervention

Docosahexaenoic acid (DHA) in the form of DHA-enriched triglyceride oil by oral administration in 400 mg/capsules x 11 per day in treatment naïve breast cancer patients who will be undergoing neoadjuvant chemotherapy.

### Known Risks and Benefits

The proposed treatment (4.4 g DHA/day) has previously been used in clinical trials (for other chronic diseases) and is not anticipated to have any adverse physiologic consequences.

A common concern with n-3 LCPUFA is the possibility of increased risk of bleeding due to anti- platelet effects, however, even when co-prescribed with anti-platelet or anti-coagulant drugs, n- 3 LCPUFA at doses up to 4 g were not associated with increased risk of bleeding ^(38; 39; 40)^.

### Justification of Treatment Regimen

The proposed experimental treatment regimen is placebo or 4.4 g DHA for approximately 12-20 weeks (not including delays). The treatment period is composed of 4-6 cycles of chemotherapy and the time up until surgery. The treatment will be administered as 1000 mg capsules containing 400 mg DHA, 3 times daily, for a total 11 capsules with meals. DHA and placebo capsules will be supplied by DSM Nutritional Products.

The proposed treatment is expected to significantly increase DHA incorporation in plasma phospholipids. The use of plasma phospholipid concentration is a good marker of status as the majority of phospholipids are synthesized in the liver and the concentration is not affected to any great extent by a recent intake of DHA (i.e. meal of salmon) or the collection of a non-fasting blood sample. If it is found to predict tumour uptake it could be a useful biomarker of DHA supplementation on tumour DHA levels. DHA incorporation into plasma phospholipids will be assessed by comparing fatty acid composition of plasma phospholipids of each subject after supplementation to their baseline fatty acid composition. This study will also compare fatty acid composition in subjects receiving DHA compared to placebo supplementation. Increased DHA incorporation has been shown to correlate with increased response to chemotherapy and overall survival in breast cancer ^(10)^. This amount of DHA (4.4 g DHA/day) was chosen because it was previously shown to be safe in other patient groups ^(41; 42; 43; 44)^. The higher dose as a proportion of dietary fat is similar to the level fed in our animal studies.

- - 1. Neoadjuvant treatment

Treatment with TCH (docetaxel/ carboplatinum/ trastuzumab) is the preferred neoadjuvant treatment at our Institution for invasive breast cancer. For this clinical trial, the dose and schedule will be based on the NCCN guideline as follows:

- - - - Docetaxel 75 mg/m2 IV day 1
      - Carboplatin AUC 6 IV day 1
        - Cycled every 21 days for 6 cycles with: Trastuzumab 8 mg/Kg IV week 1, followed by trastuzumab 6 mg/ kg IV cycled every 21 days to complete 1 year of therapy.

For triple negative patients, the preferred regimen will be as follows:

- Epirubicin -100 mg/m2 on Day 1, Fluorouracil- 500 mg/m2 on Day 1, Cyclophosphamide - 500 mg/m2 on Day 1.
  - To be repeated every 21 days x 3 cycles and followed by 3 consecutive cycles of Docetaxel (100 mg/m2) to start 21 days after final cycle of epirubicin, fluorouracil and cyclophosphamide.

## Compliance Statement

This trial will be conducted in compliance with the protocol, GCP and the applicable regulatory requirement(s).

## Description of the population to be studied

Newly diagnosed breast cancer patients undergoing neoadjuvant chemotherapy prior to definitive breast cancer surgery who have not regularly (less than 1x/week) taken supplements or consumed fortified foods containing >200mg DHA in the last 2 months.

## Trial Objectives and Purpose

**Purpose**: To evaluate incorporation of DHA in women with breast cancer in treatment naïve patients in combination with chemotherapy and to assess potential benefit of DHA in breast cancer patients. This study will reveal the potential efficacy of DHA in combination with chemotherapy, and the degree of variability of DHA incorporation in women diagnosed with breast cancer.

#### Objectives:

1. Determine the efficacy of supplemental DHA provided with standard neoadjuvant chemotherapy in patients with invasive breast cancer as measured by change in Ki67 index from biopsy to surgical excision.
2. To assess DHA incorporation into plasma phospholipids to establish the dose of DHA required to increase efficacy of chemotherapy in breast cancer patients undergoing neoadjuvant chemotherapy treatment.
3. Examine effects of DHA supplementation on immune function (plasma cytokines, chemokines and inflammatory markers and systemic immune function) in patients undergoing neoadjuvant chemotherapy
4. Identify factors that may affect DHA incorporation into plasma phospholipids.
5. Examine changes in markers for apoptosis (Caspase-3) and tumour infiltrating lymphocytes (by immunohistochemistry) following DHA supplementation in patients undergoing neoadjuvant chemotherapy.
6. Describe rate of pathological complete response in resected breast tissue and axillary nodes in breast cancer patients receiving DHA supplementation in combination with neoadjuvant chemotherapy.
7. Describe the rate of specific (see section 4.6.1) chemotherapy associated toxicities.
8. Describe the rate of hospitalizations for chemotherapy associated toxicities.
9. Assess changes in quality of life following DHA supplementation in patients undergoing neoadjuvant chemotherapy
10. Assess exercise frequency following DHA supplementation in patients undergoing neoadjuvant chemotherapy
11. Assess the rate of breast conservation, specifically the rate of lumpectomy and mastectomy.
12. Analyze the surgical reports to determine if there is a qualitative or quantitative difference between placebo and treatment arms in the volume of surgical blood loss.
13. Analyze local control, relapse free survival and overall survival by electronic medical record and / or paper medical chart review at 3, 5, and 10 years to explore possible effects on long- term outcome.

## Trial Design

Two-arm, double blind randomized control trial comparing DHA supplementation and placebo.

Figure 6: Flowchart of Trial Design with Endpoints and Proposed Experimental Analyses

## Summary of Trial Endpoints

*Further details provided in section 4.6*

#### Primary Endpoints

1. Identify changes in the prognostic biomarker Ki67 after chemotherapy at time of surgery compared to initial biopsy in breast cancer patients receiving high level of DHA supplementation in combination with chemotherapy and compared to participants receiving chemotherapy with placebo.

#### Secondary Endpoints

1. DHA incorporation into plasma phospholipids in patients receiving DHA supplementation, compared to no supplementation, in combination with chemotherapy
2. Change in systemic inflammation (CRP, IL-6, TNFα) and immune function (ability to produce IL-2 after stimulation) following DHA supplementation in combination with chemotherapy.
3. Identify additional factors that may affect DHA incorporation into plasma phospholipids.
4. Examine changes in markers for apoptosis (Caspase-3) and tumour infiltrating lymphocytes (by immunohistochemistry) following DHA supplementation in patients undergoing neoadjuvant chemotherapy.
5. Rate of pathological complete response in breast and pathological complete response in axillary nodes.
6. Rate of specific (see section 4.6.1) chemotherapy associated toxicities.
7. Rate of hospitalizations for chemotherapy associated toxicities.

#### Exploratory Analysis:

1. Assess changes in quality of life following DHA supplementation in patients undergoing neoadjuvant chemotherapy
2. Assess exercise frequency following DHA supplementation in patients undergoing neoadjuvant chemotherapy
3. Assess the rate of breast conservation, specifically the rate of lumpectomy and mastectomy.
4. Analyze the surgical reports to determine if there is a qualitative or quantitative difference between placebo and treatment arms in the volume of surgical blood loss.
5. Analyze local control, relapse free survival and overall survival by electronic medical record and / or paper medical chart review at 3, 5, and 10 years to explore possible effects on long- term outcome.

## Steps to Minimize Bias

Patients will be randomized to receive placebo or DHA (4.4 g DHA/day). Patients, pathologists, physicians, and researchers will be blinded to subject enrolment in the study and throughout trial. Blinding will only be dropped after analysis of fatty acids, systemic immune function and Ki67 is complete. All patients will consume 11 capsules/day of either placebo containing corn/soy oil blend/day or DHA.

## Trial Treatment

Patients directed to receive chemotherapy: capsules containing approximately 400 mg of DHA in the form of DHA enriched triglyceride oil or placebo (corn/soy oil blend) will be taken orally (11 capsules/day, throughout day as preferred by participant) for a total of 4.4 g DHA or placebo, for 12-20 weeks (not including delays) beginning at commencement of initial cycle of chemotherapy and continued throughout 4-6 cycles of chemotherapy (3 weeks/ cycle) up until the date of surgery. DHA or placebo will be discontinued 21-35 days after the last administration of cytotoxic chemotherapy when surgery occurs. Capsules are to be swallowed whole and not crushed or chewed.

DHA and matching corn/soy oil blend placebo capsules will be provided by DSM Nutritional Products. Capsules (DHA/placebo) in blinded bottles will be supplied to trial subjects prior to the beginning of every chemotherapy cycle by research staff. All capsules of each product supplied will be of the same brand and NPN and/or product numbers.

All subjects will be dispensed additional bottles of DHA / placebo capsules at the beginning of the study to account for circumstances where their treatment is delayed due to treatment associated toxicities (including but not limited to vomiting, diarrhea, abnormalities in blood work, fatigue or severe mouth sores). The subjects will be requested to continue taking the DHA or placebo as tolerated and will be dispensed additional capsules as necessary. The extra capsules will remain with the subject until the end of the study.

### 3.8.1 Discard/Destruction/Returns and Reconciliation

The investigator is responsible for keeping accurate records of the clinical supplies received from DSM, the amount dispensed to and returned by the subjects and the amount remaining at the conclusion of the trial. DSM will provide appropriate documentation that must be completed for drug accountability and return, or local discard and destruction if appropriate. Where local discard and destruction is appropriate, the investigator is responsible for ensuring that a local discard/destruction procedure is documented.

## Trial Duration

Subjects will take DHA or placebo capsules at start of first cycle of chemotherapy and continue DHA/placebo for 4-6, 3-week cycles of chemotherapy and up until the date of surgery. Patients will participate for a total of 12-20 weeks (not including delays). Tissue for primary and secondary endpoints will be collected at diagnosis (diagnostic core biopsy) and at time of surgical resection. Follow-up will occur as per the relevant guidelines of the Alberta Breast Cancer Program and the reporting period for adverse events will start at the time the subject takes the first dose of DHA/placebo through and including 28 calendar days after last administration of study agent. Study treatment will end when subject undergoes breast surgery (approximately 3-6 weeks after the last chemotherapy cycle).

## Withdrawal/Discontinuation Criteria

Doses of n-3 LCPUFA up to 7.56 g/day have no reported toxicity in women (9). Subjects will be discontinued from the study if serious adverse events related /possibly related to the use of DHA are reported or they are unable to continue to take at least 50% of the daily supplement over two consecutive cycles. If subjects are delayed in treatment due to treatment associated toxicities (including but not limited to vomiting, diarrhea, abnormalities in blood work, fatigue or severe mouth sores), they are requested to continue taking the DHA or placebo as tolerated. At the beginning of the study, subjects will be supplied with an additional bottle of capsules to account for any potential visit delays. These will be replaced as necessary.

A participant may also be discontinued from study treatment (but may continue to be monitored in follow-up within the trial) for any of the following reasons*:*

- Investigator’s discretion
- Disease progression
- Need for additional anticancer therapy not specified in the protocol.
- Noncompliance with study treatment or procedure requirements.
- Lost to follow up: 3 or more documented attempts to contact participant are unsuccessful.
- Sexually active participants who refuse to use medically accepted highly effective birth control methods as described in the patient selection criteria during the course of the study.
- A female participant becomes pregnant.
- Sponsor or Regulatory Agency’s discontinuation of the study.

If disease progression in the breast, nodes or distant metastatic lesions are identified or suspected during the intervention period, experimental treatment may be continued if the following criteria are met:

1. Approval from the patients treating oncologist
2. Continued ability to take oral medications
3. Continued neoadjuvant therapy with the same treatment timeline
4. Intention to proceed to breast cancer surgery following neo adjuvant therapy

Participants may withdraw from the study at any time at their own request, or they may be withdrawn at any time at the discretion of the investigator or sponsor for safety or behaviour reasons, or the inability of the participant to comply with the protocol required schedule of study visits/procedures. Permanent study discontinuation is defined as permanent cessation of study procedures.

***3.10.1 Interim Analysis***

Interim analysis will be reviewed in consultation with the CCI DSMB. After 25 evaluable patients (evaluable is defined as trial compliant subjects that have completed end of study surgery) all data and results will be submitted to the CCI DSMB for review. The DSMB will assess any possible safety concerns or marked changes in response and advise if discontinuation warranted.

## Trial Product Accountability

At the beginning of each cycle, subjects will be provided with enough capsules for the 3-week cycle and a dosing diary to record doses taken. They will be asked to bring the dosing diary back to the Clinical Trials Nurse and bottles with remaining capsules back to the Pharmacy at their next chemotherapy cycle. A new diary and bottles with enough capsules will be provided. The process will repeat until chemotherapy is completed. The Clinical Trials Nurse will review the diary and number of pills (if any) returned and record treatment compliance in the source document.

## Maintenance of Randomization

Subject will be randomized by covariate-adaptive randomization (block randomization). Subjects will be stratified by histological subtype and grade into the following categories: Luminal A, Luminal B, HER2+ and TNBC. Randomization codes will be maintained until after all data are collected and analyzed.

## Source Data

1. Subject demographics: Subject study number and initials, gender, date of birth, weight, BMI
2. Trial Data: Treatment dates (date started trial supplementation, date finished trial supplementation), number of pills taken, glucose, AST, ALP, Bilirubin, creatinine, PTT, PT/INR, CBC and differential, mammogram and ultrasound (if done) results at the time of diagnosis as well as any subsequent pre-operative imaging, ECOG Performance Status, medical history, menopause status and adverse events.
3. Primary Diagnostic Data: Method of presentation, imaging findings, date of core biopsy, surgical accession number, biomarkers on needle core (ER, PR, HER2 [IHC and SISH (if applicable)], Ki67).
4. Resection Data: Date of surgery, type of surgery, surgical accession number, biomarkers (ER, PR, HER2 [IHC and SISH (if applicable)], Ki67, histologic subtype, grade (Nuclear, Architectural, Mitotic and Overall), size, number of resected lymph nodes, number of positive nodes, lymph vascular invasion, margin status, stage.
5. Trial-specific data: Fatty acid composition of plasma phospholipids, Ki67 on core biopsy and resection specimen by image analysis. Difference between core and surgical resection Ki67 indices (absolute percentage and after log score transformation), caspase-3 and tumour infiltrating lymphocytes.
6. Quality of life and exercise frequency questionnaires.
7. Nature and incidence of specific (see section 4.6.1) toxicities related to chemotherapy.
8. Date of hospitalizations for chemotherapy-related toxicity.

# Methodology: Participants, Interventions and Outcomes

## Consent

Investigators must ensure that patients are fully informed about the purpose, potential risks, and other critical issues regarding clinical trials in which they volunteer to participate.

The Sponsor will provide an appropriate informed consent form, which will include all elements required by ICH-GCP and applicable regulatory requirements. This protocol, consent form and any amendments will be submitted to the Health Research Ethics Board of Alberta, Cancer Committee (HREBA.CC) for formal approval to conduct the study. The decision of the HREBA-CC concerning the conduct of the study will be made in writing to the investigator.

All subjects for this study will be provided a consent form describing this study and providing sufficient information for subjects to make an informed decision about their participation. The formal consent of a subject, using the HREBA-CC-approved consent form, will be obtained before that subject is submitted to any study procedure. This consent form must be signed by the subject, and the principal investigator. All patients will be informed about:

- The aims of the study
- The possible adverse events
- The procedures and possible hazards to which the patient will be exposed
- The mechanism of treatment allocation
- Strict confidentiality of any patient data
- Medical records possibly being reviewed for trial purposes by authorized individuals other than their treating physician

Study participation is voluntary and patient is free to refuse further participation in the protocol whenever he/she wants to. This will not have any impact on the patient’s subsequent care.

## Eligibility Criteria

### Inclusion Criteria

1. Women with invasive (clinical stage I, II and III) breast cancer, for whom neoadjuvant systemic therapy with chemotherapy is recommended prior to surgery.
2. ECOG Performance status of 0 or 1.
3. Hematology and Biochemistry assessments (CBC and differential, PTT, PT/INR, AST, ALP, Bilirubin, and Creatinine) within normal range unless determined not clinically significant by the qualified investigator.
4. Ability to take oral medications.
5. Adequate tissue specimen for diagnosis, biomarkers, and endpoint Ki67 assays.
   - 1. Exclusion Criteria
6. Patients undergoing surgery prior to chemotherapy.
7. Current or previous (within 2 months) daily use (>1 day/week) use of omega-3, fish oil, or other supplements or functional foods containing docosahexaenoic acid (at daily doses > 200 mg).
8. Known allergy to soy or corn.
9. Continued intake of supplements containing Vitamin C, Vitamin E or β-carotene exceeding the DRI, or other anti-oxidant supplements.
10. Symptomatic but untreated cholelithiasis.
11. History of deep venous thrombosis, active thrombophlebitis, pulmonary embolism, stroke, acute myocardial infarction, congestive cardiac failure, untreated hypertension, known inherited hypercoagulable disorder.
12. Diagnosis of any other malignancy within the previous year except for adequately treated basal cell or squamous cell skin cancer.
13. Medically documented history of a psychiatric disorder that would preclude consent
14. Partial or complete loss of vision or diplopia, from ophthalmic vascular disease.
15. Hypersensitivity to DHA or to any ingredient in the formulation or component of the container.

### Withdrawal Criteria

1. When and how: Should a subject discontinue participation in the study before the end of the study treatment, bottles containing remaining capsules and diary will be returned to the research team and the surgery will occur as scheduled.
2. The type and timing of the data to be collected for non-evaluable subjects: If chemotherapy treatment is prematurely discontinued, subjects will be withdrawn from the study. For subjects who have not completed at least 4 cycles of chemotherapy, data collection will include the number of cycles of chemotherapy and the fatty acid composition of plasma phospholipids.
3. Whether and how subjects are to be replaced: The study will continue to recruit to achieve 46 (23 per arm) evaluable subjects that have completed 4-6 cycles of DHA + chemotherapy.
4. The follow-up for subjects withdrawn from investigational product treatment/trial treatment:

For all subjects, discontinued and not, post-chemotherapy treatment or post-surgical follow- up will occur as per the relevant guidelines of the Alberta Breast Cancer Program.

## Sample Size

### Sample Size Calculation

The sample size calculation is based on the primary objective, which is to determine the efficacy of supplemental DHA provided with standard neoadjuvant as measured by change in Ki67 index from biopsy to surgical excision. Group sample sizes of 23 subjects in group one and 23 subjects in group two achieve 81% power to detect a difference between the group proportions of 0.4. The proportion in group one is assumed to be 0.3 under the null hypothesis and 0.7 under the alternate hypothesis. The proportion in group two which is control group is 0.3. The test statistic used is the two-sided t-test. The significance level of the test was targeted at 0.05 and the significance

level actually achieved by this design is about 0.0497. The dropout rate as determined from our current accrual rate which is close to 45%. Based on the current accrual rate we would need to increase the sample size to a total of 74 subjects to accommodate the high dropout rate. Hence, a total of 74 subjects (37 patients DHA supplementation, and 37 in placebo) will be required for the study. Patients who are found to be ineligible for this study after enrollment may be removed from statistical analysis and replaced at the investigators discretion. Rationale for removal will be documented in the study files

### Accrual Period

Breast cancer patients receiving neoadjuvant chemotherapy account for approximately 20% of newly diagnosed breast cancer patients, approximately 10-12/month. Assuming a conservative accrual rate of 30%, accrual of 74 subjects is estimated to be completed in 19-24 months with 3- 4 subjects enrolled per month.

## Recruitment

The breast cancer medical oncologists from the Cross Cancer Institute, all of whom are co- investigators in the study, were involved in this study design and will assist in identifying potential subjects that meet the study inclusion criteria and are interested in learning about the study. Screening of patients will be facilitated by the clinical trials nurse attending new patient rounds, where chart review of all patients can be conducted to flag patients potentially eligible for the trial. Appropriate subjects will be referred to the clinical trials nurse who will contact them to proceed with the eligibility and consent process, for potential recruitment into the study.

## 4.5 Study procedures

**STUDY CALENDAR**

| **Protocol Activity**  **(Part 1/2)** | **Screening** | **Chemotherapy** | | | | | | | | | | | | **End of**  **Treatment** | **Surgery** | **Long term**  **F-up**  **(3, 5, 10 years)** |
| --- | --- | --- | --- | --- | --- | --- | --- | --- | --- | --- | --- | --- | --- | --- | --- | --- |
|  |  | **Cycle 1** | | **Cycle 2** | | **Cycle 3** | | **Cycle 4** | | **Cycle 5** | | **Cycle 6** | |  |  |  |
| **Day** | Within 21 days before chemotherapy | D1(1) | D20 | D1 | D20 | D1 | D20 | D1 | D20 | D1 | D20 | D1 | D20 | Within 28 days after last dose |  |  |
| **Window** |  |  | + 3 days |  | + 3 days |  | + 3 days |  | + 3 days |  | + 3 days |  | + 3 days |  |  |  |
| ***Baseline documentation*** | | | | | | | | | | | | | | | |  |
| Informed Consent | X |  |  |  |  |  |  |  |  |  |  |  |  |  |  |  |
| Inclusion / exclusion criteria | X |  |  |  |  |  |  |  |  |  |  |  |  |  |  |  |
| Demographic data collection | X |  |  |  |  |  |  |  |  |  |  |  |  |  |  |  |
| Medical/oncological history (including menopausal status) | X |  |  |  |  |  |  |  |  |  |  |  |  |  |  |  |
| Height | X |  |  |  |  |  |  |  |  |  |  |  |  |  |  |  |
| Weight | X | X |  |  |  |  |  |  |  |  |  |  |  | X |  |  |
| ***Clinical assessments*** | | | | | | | | | | | | | | | |  |
| Vital Signs (2) | X | X |  | X |  | X |  | X |  | X |  | X |  | X |  |  |
| Physical Exam (2) | X | X |  | X |  | X |  | X |  | X |  | X |  | X |  |  |
| ECOG Performance Status | X | X |  |  |  |  |  |  |  |  |  |  |  | X |  |  |
| ***Questionnaires*** | | | | | | | | | | | | | | | |  |
| ESAS questionnaire  (standard of care) | X | X |  | X |  | X |  | X |  | X |  | X |  | X |  |  |
| Quality of life questionnaire | X |  |  |  |  |  |  |  |  |  |  |  |  | X |  |  |
| Godin Exercise Questionnaire | X | X |  | X |  | X |  | X |  | X |  | X |  | X |  |  |
| Food frequency questionnaire (electronic or paper) |  | X^(3)^ |  |  |  |  |  |  |  |  |  |  |  |  |  |  |
| ***Tumor analysis*** | | | | | | | | | | | | | | | |  |
| Biopsy analysis (sample previously collected): Grade/ER/PR/HER2/Ki67 (4) | X |  |  |  |  |  |  |  |  |  |  |  |  |  |  |  |
| Biopsy analysis (sample previously collected): Caspase-3, Tumour infiltrating lymphocyte | X |  |  |  |  |  |  |  |  |  |  |  |  |  |  |  |
| Surgical specimen analysis (post-intervention): Caspase-3, Tumour infiltrating lymphocytes, Ki67 (5) |  |  |  |  |  |  |  |  |  |  |  |  |  |  | X |  |

| **Protocol Activity**  **(Part 2/2)** | **Screening** | **Chemotherapy** | | | | | | | | | | | | **End of**  **Treatment** | **Surgery** | **Long term**  **F-up**  **(3, 5, 10 years)** |
| --- | --- | --- | --- | --- | --- | --- | --- | --- | --- | --- | --- | --- | --- | --- | --- | --- |
|  |  | **Cycle 1** | | **Cycle 2** | | **Cycle 3** | | **Cycle 4** | | **Cycle 5** | | **Cycle 6** | |  |  |  |
| **Day** | Within 21 days before chemotherapy | D1(1) | D20 | D1 | D20 | D1 | D20 | D1 | D20 | D1 | D20 | D1 | D20^10^ | Within 28 days after last dose |  |  |
| **Window** |  |  | + 3 days |  | + 3 days |  | + 3 days |  | + 3 days |  | + 3 days |  | + 3 days |  |  |  |
| ***Laboratory studies- standard of care*** | | | | | | | | | | | | | | | |  |
| PTT, PT/INR, AST, ALP, Bilirubin, Glucose, Creatinine | X (6) |  |  |  |  |  |  |  |  |  |  |  | X (7) |  |  |  |
| CBC and differential | X |  |  |  |  |  |  | X |  |  |  |  | X (7) |  |  |  |
| ***Laboratory studies- experimental*** | | | | | | | | | | | | | | | |  |
| Blood sample for assessment of immune function (plasma cytokines, chemokines and inflammatory markers, ex vivo measures of immune  function) (8) | X |  |  |  |  |  |  | X |  |  |  | X (7) |  |  |  |  |
| Blood sample for assessment of phospholipids (plasma phospholipid  concentration and composition) (8) | X |  |  | X |  | X |  | X |  | X |  | X | X (7) |  |  |  |
| ***Study treatment*** | | | | | | | | | | | | | | | |  |
| Treatment: DHA/Placebo (9) |  | Days 1-21 | | Days 1-21 | | Days 1-21 | | Days 1-21 | | Days 1-21 | | Days 1-21 | |  |  |  |
| Medication Diary |  | Days 1-21 | | Days 1-21 | | Days 1-21 | | Days 1-21 | | Days 1-21 | | Days 1-21 | |  |  |  |
| ***Other assessments*** | | | | | | | | | | | | | | | |  |
| Concomitant medications review | X | X |  | X |  | X |  | X |  | X |  | X |  | X |  |  |
| Adverse Events | X | X |  | X |  | X |  | X |  | X |  | X |  | X |  |  |
| Assessment of Relevant Toxicities |  | X |  | X |  | X |  | X |  | X |  | X |  | X |  |  |
| Neuropathy assessment |  | X |  | X |  | X |  | X |  | X |  | X |  |  |  |  |
| Pathological complete response |  |  |  |  |  |  |  |  |  |  |  |  |  |  | X |  |
| Breast conservation |  |  |  |  |  |  |  |  |  |  |  |  |  |  | X |  |
| Surgical blood loss assessment |  |  |  |  |  |  |  |  |  |  |  |  |  |  | X |  |
| Long term follow-up (local control, relapse free survival and overall survival) |  |  |  |  |  |  |  |  |  |  |  |  |  |  |  | X |

ESAS: Edmonton Symptom Assessment System

- - - 1. Day 1 is the first day of the chemotherapy cycle. If cycle 1 day 1 procedures were performed within 7 days as part of screening, they don’t need to be repeated unless clinically warranted.
      2. Will be repeated for each cycle of chemotherapy.
      3. Food frequency questionnaire to be completed anytime within the first cycle of chemotherapy.
      4. Physicians to request Ki67 test to be done on tumor biopsy, if not previously done as per standard of care.
      5. Analysis on surgical specimen to be performed at Dr. Bigras Laboratory, CCI.
      6. Lab done within the past 28 days does not need to be repeated unless clinically warranted.
      7. Tests required at the end of the last round of chemotherapy (i.e., end of cycle 4, 5 or 6 as per patients’ individual treatment plan).
      8. Whole blood to be collected and refrigerated at the CCI. Samples will be collected on the same day and lab work will be completed at Dr. Field’s lab, U of A.
      9. If subject’s chemotherapy is delayed due to associated toxicities, they will be encouraged to continue taking the DHA/placebo capsules as tolerated.
      10. C6D20 sample for general lab hematology/chemistry may be taken on C6D1 and the C6D20 phospholipid sample may be taken at end of treatment.
      11. Screening:

Pre-study examinations will be performed within 21 days before administration of the first dose of chemotherapy.

Screening activities outside the standard of care will only be performed after obtaining the subject’s written informed consent. The following examinations will be performed in the 21-day window prior to first chemotherapy administration:

- - - - - Signed informed consent
        - Review of inclusion / exclusion criteria
        - Demographic data collection
        - Medical/ oncological history (including menopausal status)
        - Height and weight
        - Vital signs
        - Physical examination with clinical description of primary tumour largest single palpable dimension and clinical axillary node status.
        - ECOG Performance Status assessment
        - ESAS questionnaire, as per standard of care.
        - Quality of life questionnaire.
        - Godin Exercise Questionnaire (short, 4-item self-administered questionnaire that seeks information on the number of times one engages in vigorous, moderate, light and strength/resistance exercise).
        - Standard Tumor analysis: Grade; ER/PR/HER2; Ki67 to be requested if not already performed.
        - Biopsy Tumor analysis: Caspase-3, Tumour infiltrating lymphocytes
        - Standard laboratory blood assessment (performed at any time prior to study entry): CBC and differential, PTT, PT/INR, AST, ALP, Bilirubin, Glucose, Creatinine.
        - Blood sample drawn for assessment of immune function (plasma cytokines, chemokines and inflammatory markers, ex vivo measures of immune function) and for plasma phospholipid analysis (concentration and composition).
        - Documentation of the diagnosis and disease stage.

Data collected during study (chemotherapy cycles 1-6):

- - - - - Weight (Day 1 of cycle 1 chemotherapy)
        - Vital signs
        - Physical Exam: at each cycle, clinical description of the primary tumour largest single palpable dimension and clinical axillary node status (present, absent)
        - ECOG performance status
        - Edmonton Symptom Assessment System (ESAS) questionnaire (at beginning of cycles 1-6).
        - Quality of life questionnaire (at beginning of cycle 1).
        - Exercise questionnaire (at beginning of cycles 1-6).
        - Food frequency questionnaire: Patients consenting to complete the questionnaire online, will receive a link with a password to complete it at home. Those who are not able to access online resources, will be offered a paper version (it can be completed anytime within the first cycle of chemotherapy).
        - Standard Laboratory Blood Assessment:
- CBC and differential (Day 1 of cycle 4 and end of cycle 6 or end of final chemotherapy cycle).
- PTT, PT/INR, AST, ALP, Bilirubin, Glucose, Creatinine- end of final chemotherapy cycle.
- Blood sample drawn for assessment of immune function (plasma cytokines, chemokines and inflammatory markers, ex vivo measures of immune function), Day 1 of cycle 4 and end of cycle 6 or end of final chemotherapy cycle as per patient treatment plan.
  - - - - Blood sample drawn for plasma phospholipid analysis (concentration and composition), Day 1 of each chemotherapy cycle (cycles 2-6) and end of cycle 6 or end of final chemotherapy cycle as per patient treatment plan.
        - Cytotoxic chemotherapy dose (fluorouracil/cyclophosphamide/epirubicin/doxorubicin/docetaxel/docetaxel recorded as mg administered; carboplatinum recorded as AUC) and administration date for each cycle
        - Study treatment: DHA or placebo: Days 1-21 of each cycle
        - Medication diary - days 1-21 for each cycle
        - Review of concomitant medications (at beginning of each chemotherapy cycle)
        - Adverse event reporting (beginning of each chemotherapy cycle 1-6)
        - Neuropathy assessment (at beginning of each chemotherapy cycle 1-6).
        - Assessment of compliance with study medication (at end of cycles 1-6)

End of treatment (within 28 days after last dose):

- - - - - Weight
        - Vital signs
        - Physical Exam: clinical description of the primary tumour largest single palpable dimension and clinical axillary node status (present, absent)
        - ECOG performance status
        - Edmonton Symptom Assessment System (ESAS) questionnaire
        - Quality of life questionnaire
        - Exercise questionnaire
        - Review of concomitant medications
        - Adverse event reporting
        - Neuropathy assessment, fatigue assessment, assessment of relevant toxicities and patient reported outcomes from
        - Medication diary, adverse events and compliance will be assessed.

Surgery

- Pathological Complete Response
- Breast conservation
- Assessment of surgical blood loss

Long term chart follow-up at 3, 5 and 10 years will assess local control, relapse free survival and overall survival compared between participants receiving DHA supplementation compared to placebo.

Additional testing of residual blood laboratory samples:

Consent will be obtained to store samples not used, in a bio-bank for future nutrient, metabolic or biomarker analysis. This will be described in the ethics submission and consent form.

- - 1. ***Measures taken to minimize/avoid bias***
       1. Blinding

A placebo capsule containing a blend of corn and soy oil will be manufactured to be the same size, shape, and colour as the DHA capsules. Bottles with DHA/placebo capsules should be stored between 15-25 ºC.

- - - 1. Randomization

A biostatistician will generate a patient randomization list and send it to an unblinded Clinical Trials Coordinator (Clinical Trials Unit), who will be alerted when a patient is ready to be randomized and will be responsible for assigning the treatment number from the applicable arm (based on pharmacy stock) and providing the information to the blinded study staff (Clinical Trials Nurse/ Clinical Trials Coordinator) confirming the unique study identifier and treatment #.

The Clinical Trials Nurse will then notify Pharmacy staff, who will dispense the appropriate treatment at day 1 of each chemotherapy cycle. Patients should be given a sufficient supply to last until their next study visit. Following each randomization, the unblinded Clinical Trials Coordinator will keep details on patient E#, Study ID # and Randomization group, covered and placed inside a sealed envelope for the PI to use in case there is an urgent need for breaking of the blind.

4.5.3.3. Breaking the blind

A separate database is available for access by selected staff (Principal Investigator and lead Clinical Trial Nurse), in case there is a need for breaking the study blind. The study blind should only be broken in emergency situations for patient safety. When a blinding code is broken, the date and reason for unblinding must be fully documented in source documents and entered on the case report form. Every effort should be made by site staff to ensure that the treatment arm in which the unblinded patient is assigned is communicated only to those site staff that require the information for treatment purposes.

- - 1. ***Medications/Treatments Permitted***

Medication(s)/treatment(s) permitted (including rescue medication) before and/or during the trial include any not stated in 4.5.4.

### Medications/Treatments Prohibited/Restricted

- - - 1. Current or previous (within 2 months) daily use (>1 day/week) use of omega-3, fish oil, or other supplements or functional foods containing docosahexaenoic acid (at daily doses > 200 mg).
      2. Continued intake of supplements containing Vitamin C, Vitamin E or β-carotene exceeding the DRI (Daily recommended intake), or other anti-oxidant supplements. These will be discussed with each subject.

## Outcomes, Methodology and Assessment

### Primary Outcomes

#### Change in Ki67 at biopsy to surgical excision

**Description:** Ki67 changes will be calculated by image analysis and will follow analytical and pre-analytical recommendations of Dowsett et al. ^(33)^.

**Time Frame:** Ki67 will be measured at baseline biopsy and at experimental end (surgical excision). Ki67 assays will be performed and reported as part of the routine diagnostic services.

**Methods:** Ki67 will be tested by immunohistochemistry (IHC) by the diagnostic biomarker laboratory at the Cross Cancer Institute using the MIB1 antibody on 4 µm sections from formalin fixed paraffin embedded (FFPE) needle core biopsy surgical specimens.

**Ki67 scoring methodology**: Dr. Gilbert Bigras, CCI breast pathologist, has developed a semi- automated computer algorithm scoring system to give objective and reproducible Ki67 scores using this method: [(https://www.ncbi.nlm.nih.gov/pubmed/27093453)](https://www.ncbi.nlm.nih.gov/pubmed/27093453) to quantitate Ki67 scoring for all study subjects. This method is well suited to assess "hot proliferative tumoral spots". Furthermore, all KI67 IHC preparations will also be assessed using QuPath protocol for Ki67 IHC evaluation on whole slide ([https://github.com/qupath)](https://github.com/qupath) which will provide an integrated value for the entire tumour. At final analyses (after 46 evaluable subjects), Ki67 staining will be repeated as single IHC stain and interpreted by image analysis. In addition, the original single stained slides will be interpreted visually by research staff. At time of Ki67 interpretation, slides will be de-identified and coded to prevent potential bias.

**Reliability and Validity of Analysis:** The Ki67 index is validated and used in clinic as marker of proliferation. The Ki67 index (absolute % and log transformed) of biopsy and surgical resection (after chemotherapy) will be compared on each participant, and 95% t-confidence interval for mean percent change in Ki67 from baseline will be compared between participants receiving DHA compared to placebo. Data will be analyzed at completion of trial (74 subjects).

All Ki67 values (routine and image analysis) will be recorded as absolute percentage and as log-transformation in REDCap trial database and participant’s case report form.

### Secondary Outcomes

#### Changes in level of DHA incorporation in plasma phospholipids

**Description:** Changes in level of DHA incorporation in plasma phospholipids will be assessed to identify the range of DHA incorporation in this patient population. From our hypothesis and previously published data, we predict that incorporation will be heterogeneous in our patient population. We further expect that giving the 4.4g DHA/day will result in a significant increase in DHA incorporation in all subjects. We will also examine if disease subtype or stage affects DHA incorporation into plasma. If possible with the small study size, we will also assess difference in DHA incorporation in subjects with different breast cancer subtypes. The goal is to determine if plasma phospholipids can be used to predict treatment outcome.

**Time Frame:** Plasma phospholipids will be measured at screening, Day 1 of each cycle of chemotherapy (cycles 2-6) and end of cycle 6 or end of final chemotherapy cycle as per patient treatment plan. The whole blood sample will be stored at room temperature for 20 minutes at the CCI lab and then stored in the fridge at 4˚C until pick-up. All analysis will be completed at the U of A Nutritional Immunology laboratory (Dr. Field’s lab).

**Methods:** At the U of A: Venous blood will be centrifuged at 900 g for 10 min to obtain plasma. Plasma will be separated in 6 aliquots, and immediately frozen at -70°C for storage. Plasma (concentration and relative percent) will be extracted by modified Folch procedure^(45)^, phospholipids separated by thin layer chromatography and fatty acid content measured by gas- liquid chromatography as previously described ^(46; 47)^.

**Reliability and Validity of Analysis:** The percentage change in DHA from baseline will be compared in each subject and a 95% t-confidence interval for the mean percent change in the DHA from baseline will be compared to subjects receiving placebo. An internal standard is used to identify and quantify the fatty acid. This is a standard measure for fatty acid status has coefficient of variation <5% and individual GC peaks are validated against phospholipid standards from NuChek. All fatty acid composition will be recorded in REDCap trial database.

#### Systemic immune function

**Description:** Systemic immune function will be assessed on stored plasma by measuring plasma chemokines, cytokines and inflammatory markers in peripheral blood samples. Changes in these markers will be assessed in each subject compared to baseline levels. To determine the effect of DHA on immune function before and after DHA supplementation, buffy coats (from the plasma separation) will be obtained from the sample at the three timepoints. We will also compare changes in subjects given DHA supplements and placebo, and examine the relationship between changes in specific markers and the level of DHA incorporation. In addition, we will measure the individual cell types and their activation state by flow cytometry using specific mAb against cell surface proteins.

**Time Frame:** Effect of DHA supplementation on immune function will be assessed at screening, Day 1 of cycle 4 and at the end of cycle 6 or end of final chemotherapy cycle as per patient treatment plan in immune cells isolated from blood following supplementation compared to baseline, and compared to subjects receiving placebo. Whole cell blood analysis of immune cell subsets and isolation of peripheral mononuclear cells will be done immediately on peripheral blood samples once they arrive in the PI laboratory. Cytokines, chemokines and inflammatory markers in plasma aliquots and cytokines in supernatant of cultured lymphocytes will be analyzed when all samples have been obtained.

**Methods:** Previous research has determined that blood stored on ice can be stored for up to 12 hours prior to isolation. Peripheral mononuclear cells will be isolated on a Ficoll density gradient of Histopaque 1077 as previously described ^(49; 50)^. Whole blood phenotyping of the immune cell types will be identified using specific fluorescently labelled mAb to surface receptors. These will be quantified by flow cytometry, as described ^(50; 51)^. To measure cytokine production in isolated lymphocytes, cells will be cultured in media with or without Phytohemagglutinin (PHA) or lipopolysaccharide (LPS) for 48 h. Supernatant will be collected and stored at -80°C for cytokine analysis. Cytokines will be measured using electrochemiluminescent multiplex assays (MesoScale Discovery; as previously described ^(48)^) or by individual ELISA assays. Cytokines, chemokines and inflammatory markers in plasma aliquots and cytokines in supernatant of cultured lymphocytes will be analyzed when all samples have been obtained. All data will be entered and maintained in REDCap trial database.

**Reliability and Validity of Analysis:** Changes in systemic immune function will be assessed in subjects compared to baseline, and compared to subjects in placebo group. The data analysis will occur at completion of trial. Cytokines are done in duplicate and the coefficient of variance is <15%.

#### Additional Factors affecting DHA incorporation into plasma phospholipids

**Description**: If incorporation of DHA in plasma phospholipids is variable within the DHA treatment arm, possible factors that may influence incorporation will be assessed between high and low incorporators.

**Time Frame**: Additional parameters will be assessed at end of study from samples collected throughout the study.

**Methods:** Possible factors that predict incorporation, including, including weight (BMI), age, the usual diet of the women (estimated from the FFQ), composition of fat in the women’s usual diet (estimated from the FFQ), histology of the tumour (provided from the biopsy) and amount of DHA consumed (compliance to the supplement) will be assessed after calculating high and low DHA incorporators.. Analysis will be carried out after final analysis of DHA incorporation and data will be entered and maintained in REDCap trial database.

#### Changes in markers of apoptosis and tumour infiltrating lymphocytes following

#### DHA supplementation

**Description:** Caspase-3 changes and tumour infiltrating lymphocyte presence will be calculated by image analysis and a comparison of treatment to placebo will be made.

**Time Frame:** Caspase-3 and tumour infiltrating lymphocyte will be measured at baseline biopsy and at experimental end (surgical excision).

**Methods:** Caspase-3 and tumour infiltrating lymphocyte changes will be tested by immunohistochemistry (IHC) by the diagnostic biomarker laboratory at the Cross Cancer Institute on 4 µm sections from formalin fixed paraffin embedded (FFPE) surgical specimens.

**Caspase-3 and tumour infiltrating lymphocyte** **scoring methodology.** At final analyses (after 46 evaluable subjects), IHC staining will be interpreted by image analysis. At time of interpretation, slides will be de-identified and coded to prevent potential bias. Proportions of positively stained tumour cells will be scored by two pathologists and the staining intensity will be recorded independently. Proportions of negative cells, weakly positive cells and strongly positive cells will be estimated.

**Reliability and Validity of Analysis:** Caspase-3 is a validated marker of apoptosis and tumour infiltrating lymphocytes mark immune infiltration in the tumour. The calculated indices (absolute % and log transformed) of surgical resection (after chemotherapy) will be compared between participants receiving DHA and the placebo group, and 95% t-confidence interval for mean percent change will be determined. Data will be analyzed at completion of trial.

#### Pathological complete response rate

**Description**: Pathological complete response in resected breast tissue and all sampled axillary nodes will be assessed as absence of invasive cancer. Pathologic complete response will be classified as ypT0/is ypN0.

**Time Frame**: Pathological complete response will be determined at end of study after surgical resection as part of standard of care assessment.

**Methods:** Resected breast tissue and axillary nodes will be assessed in hematoxylin and eosin stained tissue for evidence of invasive disease according to standard of care.

**Reliability and Validity of Analysis:** The rate of pathological complete response in breast tissue and axillary nodes after surgical resection will be compared between participants receiving DHA supplementation compared to placebo. Data will be recorded in patient’s case report form, entered into REDCap trial database analyzed at completion of the trial.

#### Comparison of rate of specific chemotherapy associated toxicities between treatment arms

**Description:** Rate of specific chemotherapy associated toxicities, and chemotherapy- associated hospitalizations will be compared between DHA and placebo arms. This study will focus on the following toxicities: Peripheral Sensory/Motor Neuropathy (Grades 1, 2, 3, 4, 5). Arthralgia (Grade 3), Myalgia (Grade 3), Fatigue (Grade 3) and the presence of Febrile Illness/Infection in the last cycle. Any changes will then be examined in regards to level of supplementation and DHA incorporation.

The study will also collect any adverse events that may be related or are related to the DHA/Placebo for safety considerations.

**Time Frame**: These analyses will be completed at end of study after surgical resection.

**Methods:** Toxicities will be assessed by the clinical trial nurse on day 1 of each chemotherapy cycle. Dates of hospitalization will be recorded in patient’s case report form and REDCap trial database.

**Reliability and Validity of Analysis:** Rates of specific chemotherapy-associated toxicities, all grade neuropathy and hospitalizations will be compared between DHA supplementation and placebo arms as scored by a medical oncologist in a standardized toxicity/ neuropathy form for each cycle of chemotherapy.

**Other Pre-specified Outcome Measures**

Exploratory analysis:

1. **Quality of Life**

**Description**: Assessment in changes in quality of life will be determined by questionnaire employed at baseline and end of treatment.

**Time Frame**: These analyses will be completed at end of study.

#### Methods: Changes in quality of life will be determined by assessment of questionnaires at the end of study. Comparisons will be assessed from end of treatment to baseline within and between treatment groups

**Reliability and Validity of Analysis**: The quality of life questionnaire is based on a validated questionnaire from European Organization for Research and Treatment of Cancer- Quality of Life Questionnaire-C30 (EORTC-QLQ-C30)**.**

#### Exercise frequency

#### Description: Assessment of exercise frequency will be determined by questionnaire employed at baseline, beginning of each cycle of chemotherapy and end of treatment.

#### Time Frame: These analyses will be completed at end of study.

#### Methods: The frequency and intensity of exercise will be assessed using the modified Godin Leisure-Time Exercise Questionnaire (GLTEQ) at baseline and beginning of each chemotherapy cycle. Comparisons will be assessed from end of treatment to baseline within and between treatment groups.

**Reliability and Validity of Analysis**: The Godin Leisure-time Exercise questionnaire is a validated questionnaire (Godin, G.; Shephard, R.J. Godin leisure-time exercise questionnaire. *Medicine & Science in Sports & Exercise* **1997**, *26* S36-S38).

#### The rate of breast conservation

**Description**: The rate of breast conservation, specifically the rate of lumpectomy and mastectomy.

**Time Frame**: These analyses will be completed at end of study after surgical resection.

**Methods:** The rate of breast conservation, will be determined by surgical and pathologic reports at time of surgical resection, recorded in the patient’s care report form and entered into REDCap trial database.

**Reliability and Validity of Analysis:** The frequency of breast conservation will be compared between participants receiving DHA supplementation compared to placebo. Data will be analyzed at completion of the trial and validated by a medical oncologist.

#### The volume of surgical blood loss

**Description**: DHA is variably reported to increase bleeding time.

**Methods**: Volume estimates of blood loss will be assessed by review surgical report estimates of blood loss to see if there is a qualitative or quantitative difference between placebo and treatment arms, once adjusted for the magnitude of surgery (lumpectomy vs. mastectomy vs. mastectomy

+ immediate reconstruction; sentinel node dissection vs. full axillary dissection).

#### Local control, relapse free survival and overall survival

**Description:** Local control, relapse free survival and overall survival will be analyzed by review of electronic medical records, registry reports, and / or paper medical charts.

**Methods:** Local control, relapse free survival and overall survival will be analyzed by electronic medical record and / or paper medical chart review at 3, 5, and 10 years.

**Reliability and Validity of Analysis:** Local control, relapse free survival, and overall survival will be compared between participants receiving DHA supplementation compared to placebo. Data will be validated by a medical oncologist.

# Methodology: Data Collection, Management, and Analysis

## 5.1 Data Collection Methods

### 5.1.1 Case Report Forms (CRFs)

Validated REDCap will be used for the Case Report Forms.

## Statistics

### Description of Statistics

*Primary Outcome*:

1. Ki67: The percent change in Ki67 will be determined as an absolute percentage. The number of patients showing a decrease and the 95% confidence interval for the mean percent change in the Ki67 level from baseline in subjects receiving DHA supplementation will be compared to subjects receiving placebo. Test of proportions will be used to compare the proportions between the two groups.

*Secondary Outcomes*:

- 1. DHA incorporation: The Wilcoxon signed rank test will be used to compare the plasma DHA level at each cycle of chemotherapy with baseline. The difference in DHA incorporation from baseline will be calculated and the 95% confidence interval for the mean percent change in DHA level from baseline will be assessed. The difference between

% DHA in plasma phospholipids between DHA supplementation and placebo arms will be calculated, and the 95% confidence interval will be reported.

- 1. Systematic immune function: Changes in inflammatory markers and immune function will be analyzed. If data is not normally distributed it will be log transformed prior to analysis and the normality assumptions will be tested again. Repeated measures ANOVA with post hoc analysis will be used to determine if there is an effect of treatment on immune function.
  2. Factors affecting DHA incorporation: Factors affecting DHA incorporation will be examined by multivariate analysis. The outcome of interest is binary (DHA vs. Placebo); hence binary logistic regression will be used to determine the factors associated with the outcome variable. Factors significant at the univariate analysis will be entered into the multivariate model. Odds ratio and the corresponding 95% confidence interval will be reported.
  3. Caspase-3 and tumour infiltrating lymphocytes: The percent change in these markers will be determined as an absolute percentage. The 95% confidence interval for the mean percent change in these levels in subjects receiving DHA supplementation will be compared to subjects receiving placebo. Test of proportions will be used to compare the proportions between the two groups.
  4. Pathological complete response: The number of patients showing a pathological complete response in breast or axillary nodes will be examined, and the 95% confidence interval for the mean percent change in pathological complete response in subjects receiving DHA supplementation will be compared to subjects receiving placebo.
  5. Rate of specific (see section 4.6.2) chemotherapy associated toxicities and hospitalization: The number of chemotherapy associated adverse events and hospitalizations will be compared and 95% confidence interval for the mean percent change in events in subjects receiving DHA supplementation will be compared to subjects receiving placebo.

SAS version 9.4 (SAS Institute Inc., Cary, NC) software will be used for statistical analysis. A p-value <0.05 level will be used for all statistical significance. Two-sided tests will be used for all statistical tests.

*Exploratory analysis:*

1. Assessment in changes in quality of life and frequency and intensity of exercise will be assessed from time points collected to baseline within and between treatment groups. Analyses of covariance will be assessed to compare differences between treatment arms
2. The degree of breast conservation will be compared between participants receiving DHA supplementation compared to placebo. Chi-square tests will be used to compare the degree of breast conservation between the two study arms. Data will be analyzed at completion of the trial.
3. The volume of surgical blood loss will be compared between participants receiving DHA supplementation compared to placebo. Chi-square tests will be used to compare the degree of breast conservation between the two study arms. Data will be analyzed at completion of the trial.
4. Local control, recurrence free survival, and overall survival: Rate of local control will be compared between participants receiving DHA supplementation compared to placebo using t- test of proportions. Recurrence-free survival and survival will be analyzed using the log rank test on Kaplan-Meier survival curves.

SAS software, version 9.4 (SAS Institute Inc., Cary, NC), will be used for statistical analysis. A p-value <0.05 level will be used for all statistical significance. Two-sided tests will be used for all statistical tests

## Criteria for the Termination of the Trial

The investigator has the right to close the study at any time. These decisions will be taken in consultation or in discussion with the CCI Data Safety Monitoring Board (DSMB).

The sponsor (AHS-CCI) has the right to close this study at any time, which may be due but not limited to the following reasons:

### Futility

If the study conduct (e.g. recruitment rate, drop-out rate, data quality, protocol compliance) does not suggest a proper completion of the study within a reasonable time frame the trial will be terminated.

### Safety

### Trial will be terminated if a significant increase in SAEs is identified by the medical oncologists. The DSMB will assess any possible safety concerns or marked changes in response and advise if study should be terminated for safety concerns.

### If risk-benefit ratio becomes unacceptable owing to, for example,

- - - - Safety findings from this study (e.g. SAEs);
      - Results of any interim analysis
      - Results of parallel clinical studies
      - Results of parallel animal studies (e.g. toxicity, teratogenicity, carcinogenicity or reproduction toxicity).

### Efficacy

In case of a partial study closure, ongoing subjects, including those in post study follow-up, must be taken care of in an ethical manner. If the results of any interim analysis demonstrate the primary objective has been attained further accrual would be unethical and the study will be closed.

For any of the above closures, the following applies:

- Closures should occur only after consultation between involved parties.
- All affected institutions (e.g. REB; competent authorities; CCI) must be informed as applicable according to local law. Health Canada should be notified.
- All study materials (except documentation that has to remain stored at site) must be returned to the sponsor. The investigator will retain all other documents until notification given by the sponsor for destruction.
- In case of a partial study closure, ongoing subjects, including those in post study follow-up, must be taken care of in an ethical manner.

## Deviations

The investigator is responsible to identify, document, assess and report all protocol deviations to the Sponsor and REB in accordance with Sponsor’s and REB’s requirements.

## Data Management

All data will be entered and maintained in a validated REDCap trial database. Direct access to clinical and laboratory information on the enrolled trial subjects will be limited to the principal investigator, co-investigators, trainees/staff in Dr. Field’s laboratory who have had the appropriate training and approval and study nurses and study coordinators who will have access to the source documents through the electronic medical record and laboratory information system. These personnel are aware of the specifics of the Alberta Health Information Protection legislation and have taken courses in preserving patient confidentiality as mandated by Alberta Health Services.

### Data Accounting

All subjects will have biopsy and tumor samples for analysis and we do not expect any missing data for the primary endpoint (Ki67). If treatment compliance is below 50% for 2 sequential chemotherapy cycles, or participants do not complete chemotherapy or have surgery with no neo-adjuvant treatment, will be excluded from final analysis.

If subjects do not have sufficient blood samples for the secondary analyses (DHA incorporation, systemic immune function), then this data will be omitted, and analysis will be performed using data from the remaining subjects.

### Confidentiality of Trial Documents and Patient Records

The investigators must assure that patients’ anonymity will be maintained and that their identities are protected from unauthorized parties. On CRFs, patients should not be identified by their names, but by an identification code. Investigators should keep patients’ written consent forms (original) and a patient enrolment log at the site showing codes, names and addresses.

### Retention of Patient Records and Study Files

The investigator must retain all study records and source documents for the maximum period required by applicable regulations and guidelines, or institution procedures, whichever is longer.

# Quality Control, Quality Assurance and Monitoring

## Source Data, Documents, and Monitoring

The Principal Investigator will maintain accurate source records from with case report forms are based. The investigator agrees to allow the monitor direct access to all relevant documents. In accordance with applicable regulations, GCP, and sponsor’s procedures, this study will be monitored as appropriate to assess compliance with the protocol and to assess the quality and integrity of the data being collected. When reviewing data collection procedures, the discussion will also include identification and documentation of source data items.

The sponsor / designee will monitor the site activity to verify that the:

- - - The rights and wellbeing of human subjects are protected.
    - The reported trial data are accurate, complete, and verifiable from source documents.
    - The conduct of the trial is in compliance with the currently approved protocol/amendment(s), with GCP, and with applicable regulatory requirements(s).

## Data Monitoring

### Data Safety Monitoring Board

The trial activities performed at the CCI will be monitored by the Cross Cancer Institute, Investigator Initiated Trials Data Safety Monitoring Board (DSMB). The DSMB is independent of the investigator and is composed of representatives from both medical and radiation oncology. Further details about its charter can be found by contacting the Project Manager, Investigator Initiated Clinical Trials at the Cross Cancer institute.

- - - 1. *Assessment of Safety*

##### Safety Parameters

Subjects will be assessed prior to beginning trial. DHA at up to 7.5 g/day has previously been shown to be safe with only minor GI complaints reported in a few subjects. After 25 evaluable patients, all data and results will be submitted to the DSMB for review. The DSMB will assess any possible safety concerns or marked changes in response and advise if discontinuation warranted.

##### Reporting and Recording Adverse Events

The investigator will assess the relationship between protocol treatment and the occurrence of AEs and this assessment will be recorded in the database for adverse events. This study will use the International Common Terminology Criteria for Adverse Events (CTCAE), version 5.0, for adverse event reporting. The reporting period for adverse events will start at the time the subject takes the first dose of DHA/placebo through and including 28 calendar days after last administration of study agent.

##### Follow up of Adverse Events

The reporting period for adverse events will start at the time the subject takes the first dose of DHA/placebo through and including 28 calendar days after last administration of study agent.

##### Serious Adverse Events

Serious adverse events (SAE) as defined by the Good Clinical Practice Guideline is any untoward medical occurrence that at any dose:

- Results in death
- Is life-threatening (defined as an event in which the subject was at risk of death at the time of the event; it does not refer to an event which hypothetically might have caused death if it were more severe)
- Requires inpatient hospitalization or prolongation of existing hospitalization
- Results in persistent or significant disability/incapacity
- Is a congenital anomaly/birth defect
- Is an important medical event (defined as a medical event(s) that may not be immediately life-threatening or result in death or hospitalization but, based upon appropriate medical and scientific judgment, may jeopardize the subject or may require intervention to prevent one of the other outcomes listed in the definition above.

Examples of such events are intensive treatment in an emergency room or at home for allergic bronchospasm; blood dyscrasias or convulsions that do not result in hospitalization; or development of drug dependency or drug abuse.

The PI will be made aware of all Serious Adverse Events within 24 hours. All SAEs will be reported to the local research ethics board and regulatory authorities, as applicable, in accordance with local guidelines. If serious adverse reaction to treatment occurs, the Natural and Non- prescription Health Products Directorate (NNHPD) Clinical Trial Unit and Health Ethics Research Board of Alberta, Cancer Committee (HREBA.CC) and DSM will be notified as per guidelines. It is unlikely that an adverse event in this trial will be due to the Natural Health Product.

[Section 78 of the *NHP Regulations*, ICH Guidance Document *E2A: Clinical Safety Data Management: Definitions and Standards for Expedited Reporting*].

If the serious unexpected adverse reaction is fatal or life threatening, the NNHPD should be notified immediately if possible, and no later than seven days after the sponsor becomes aware of the information

If the serious unexpected adverse reaction is neither fatal nor life threatening, the NNHPD should be notified immediately if possible, and no later 15 days after the sponsor becomes aware of the information

Within eight days after having informed the NNHPD of a serious unexpected adverse reaction to the NHP, the sponsor must submit a report as complete as possible that includes an assessment of the importance and implication of any findings. The final report should include relevant previous experience with the same or similar health products.

Serious adverse events unrelated to the study treatment do not require expedited reporting to the NNHPD. If cause of adverse event is uncertain, report will be submitted as per S ICH Guidance Document [*E2A: Clinical Safety Data Management: Definitions and Standards for Expedited*](http://www.ich.org/) [*Reporting*.](http://www.ich.org/)

Each serious unexpected adverse reaction will be reported individually in accordance specification in ICH Guidance Document *E2A: Clinical Safety Data Management: Definitions and Standards for Expedited Reporting.*

## 6.3 Auditing

Clinical trial audits provide assurance that the rights, safety and wellbeing of patients are properly protected, to assess compliance with the protocol, processes and agreements, ICH GCP standards and applicable regulatory requirements, and to assess the quality of data.

The investigator(s)/institution(s) will permit trial-related monitoring, audits, REB, DSMB review, and regulatory inspection(s), providing direct access to paper and/or electronic documentation pertaining to the clinical study (e.g. CRFs, source documents such as hospital patient charts and investigator study files). All site facilities related to the study conduct could be visited during an audit (e.g. pharmacy, laboratory, outpatient department). The investigator agrees to co-operate and provide assistance at reasonable times and places with respect to any auditing activity.

# Approvals

## Ethics

This study will be submitted to the Health Research Ethics Board of Alberta – Cancer Committee (HREBA.CC) for approval.

### Patient Protection

The responsible investigator will ensure that this study is conducted in compliance with the protocol and in agreement with the Declaration of Helsinki.

The protocol has been written, and the study will be conducted according to the ICH Harmonized Tripartite Guideline on Good Clinical Practice. Applicable government regulations, NNHPD clinical trial guidelines and Alberta Health Services research policies and procedures will also be followed.

A Clinical Trial Application (CTA) will be submitted to the Natural and Non-prescription Health Products Directorate (NNHPD). A Notice of Authorization is required prior to starting any study- related activities.

The Health Research Ethics Board of Alberta – Cancer Committee (HREBA.CC) must approve the protocol, Informed Consent Form and any trial materials given to participants.

All potential serious breaches of GCP must be reported to Sponsor or designee immediately. A serious breach is a breach of the conditions and principles of GCP in connection with the study or the protocol, which is likely to affect, to a significant degree, the safety or physical or mental integrity of the subjects of the study or the scientific value of the study.

### Subject Identification

A sequential identification number will be automatically allocated to each patient registered in the trial. This number will identify the patient and will be included on all case report forms (CRFs).

# Protocol Amendments

Before study initiation, the investigator will have written and dated approval/favourable opinion from the IRB/IEC for the protocol, consent form, subject recruitment materials (e.g., advertisements), and any other written information to be provided to subjects. The investigator will also provide the IRB/IEC with a copy of the Investigator Brochure, Product Monograph, etc. (as applicable) or product labeling information to be provided to subjects and any updates. The investigator, Sponsor or designee will provide the IRB/IEC with reports, updates and other information (e.g., expedited safety reports, amendments, and administrative letters) according to regulatory requirements or institution procedures.

# Financing and Insurance

Financing support is being provided from DSM Nutritional Products USA (Head office Netherlands) to provide the DHA supplements and the placebo. Financial support to fund the

immune analysis in Dr. Field’s laboratory at the University of Alberta, has been obtained through a CIHR project grant. This includes funding for trainees and research assistant salaries and for the proposed immune measures. Investigator Initiated Trial Funding has also been secured through the Alberta Cancer Foundation.

# Protocol Registration

The sponsor has committed to the global industry position on disclosure of information about clinical trials. The International Committee of Medical Journal Editors (ICMJE) also requires trial registration as a condition of the publication of research results generated by a clinical trial. The information regarding this trial will be made publicly available on the internet at [www.clinicaltrials.gov.](http://www.clinicaltrials.gov/)

# Publication Policy

The publication of the main trial results will be written by the Principal Investigator on the basis of the final analysis and will be sent to a major scientific journal. Authors of the manuscript will include at least the Principal Investigator and any co-investigators who have i) included eligible patients in the trial (by order of inclusion) or ii) contributed significantly to the design, conduct and data interpretation regarding companion basic science studies

Publication or presentation of study data before the publication of the primary trial endpoint may be authorized at the discretion of the Principal Investigator. The data collected during this study are confidential.

## 12 References

1. Burdge GC, Wootton SA (2003) Conversion of alpha-linolenic acid to palmitic, palmitoleic, stearic and oleic acids in men and women. In Prostaglandins, leukotrienes, and essential fatty acids

Prostaglandins Leukot Essent Fatty Acids, vol. 69, pp. 283-290.

2. Clandinin MT, Cheema S, Field CJ et al. (1991) Dietary fat: exogenous determination of membrane structure and cell function. FASEB J 5, 2761-2769.

3. Stillwell WaW, SR (2003) Docosahexaenoic acid: Membrane properties of a unique fatty acid. Chem Phys Lipids 126, 1-27.

4. Ewaschuk JB, Newell M, Field CJ (2012) Docosahexanoic acid improves chemotherapy efficacy by inducing CD95 translocation to lipid rafts in ER(-) breast cancer cells. Lipids 47, 1019-1030.

5. Chauvin L, Goupille C, Blanc C et al. (2016) Long chain n-3 polyunsaturated fatty acids increase the efficacy of docetaxel in mammary cancer cells by downregulating Akt and PKCepsilon/delta-induced ERK pathways. Biochim Biophys Acta 1861, 380-390.

6. Manni A, Richie JP, Jr., Xu H et al. (2014) Influence of omega-3 fatty acids on Tamoxifen-induced suppression of rat mammary carcinogenesis. International journal of cancer 134, 1549-1557.

7. Mason JK, Klaire S, Kharotia S et al. (2015) α-linolenic acid and docosahexaenoic acid, alone and combined with trastuzumab, reduce HER2-overexpressing breast cancer cell growth but differentially regulate HER2 signaling pathways. Lipids in Health and Disease 14, 91.

8. Bougnoux P, Germain E, Chajes V et al. (1999) Cytotoxic drugs efficacy correlates with adipose tissue docosahexaenoic acid level in locally advanced breast carcinoma. Br J Cancer 79, 1765-1769.

9. Yee LD, Lester JL, Cole RM et al. (2010) Omega-3 fatty acid supplements in women at high risk of breast cancer have dose-dependent effects on breast adipose tissue fatty acid composition. Am J Clin Nutr 91, 1185-1194.

10. Bougnoux P, Hajjaji N, Ferrasson MN et al. (2009) Improving outcome of chemotherapy of metastatic breast cancer by docosahexaenoic acid: a phase II trial. Br J Cancer 101, 1978-1985.

11. Schley PD, Jijon HB, Robinson LE et al. (2005) Mechanisms of omega-3 fatty acid-induced growth inhibition in MDA-MB-231 human breast cancer cells. Breast Cancer Res Treat 92, 187-195.

12. Blanckaert V, Kerviel V, Lepinay A et al. (2015) Docosahexaenoic acid inhibits the invasion of MDA‑MB‑231 breast cancer cells through upregulation of cytokeratin‑1. International Journal of Oncology 46, 2649-2655.

13. Li CC, Yao HT, Cheng FJ et al. (2015) Docosahexaenoic Acid Downregulates EGF-Induced Urokinase Plasminogen Activator and Matrix Metalloproteinase 9 Expression by Inactivating EGFR/ErbB2 Signaling in SK-BR3 Breast Cancer Cells. Nutrition and cancer 67, 771-782.

14. Yun EJ, Song KS, Shin S et al. (2016) Docosahexaenoic acid suppresses breast cancer cell metastasis by targeting matrix-metalloproteinases. Oncotarget 7, 49961-49971.

15. Tisza MJ, Zhao W, Fuentes JS et al. (2016) Motility and stem cell properties induced by the epithelial-mesenchymal transition require destabilization of lipid rafts. Oncotarget 7, 51553-51568.

16. Xiong A, Yu W, Liu Y et al. (2016) Elimination of ALDH+ breast tumor initiating cells by docosahexanoic acid and/or gamma tocotrienol through SHP-1 inhibition of Stat3 signaling. Molecular carcinogenesis 55, 420-430.

17. Calder PC (2015) Marine omega-3 fatty acids and inflammatory processes: Effects, mechanisms and clinical relevance. Biochim Biophys Acta 1851, 469-484.

18. Janakiram NB, Mohammed A, Rao CV (2011) Role of lipoxins, resolvins, and other bioactive lipids in colon and pancreatic cancer. Cancer metastasis reviews 30, 507-523.

19. Kim YS, Sayers TJ, Colburn NH et al. (2015) Impact of dietary components on NK and Treg cell function for cancer prevention. Molecular carcinogenesis 54, 669-678.

20. Janakiram NB, Mohammed A, Madka V et al. (2016) Prevention and treatment of cancers by immune modulating nutrients. Molecular nutrition & food research 60, 1275-1294.

21. Hajjaji N, Besson P, Bougnoux P (2012) Tumor and non-tumor tissues differential oxidative stress response to supplemental DHA and chemotherapy in rats. Cancer Chemother Pharmacol 70, 17-23.

22. D’Eliseo D, Velotti F (2016) Omega-3 Fatty Acids and Cancer Cell Cytotoxicity: Implications for Multi-Targeted Cancer Therapy. Journal of Clinical Medicine 5, 15.

23. Newell M, Brun M, Field CJ (2019) Treatment with DHA Modifies the Response of MDA-MB-231 Breast Cancer Cells and Tumors from nu/nu Mice to Doxorubicin through Apoptosis and Cell Cycle Arrest. The Journal of Nutrition, nxy224-nxy224.

24. Menendez JA, Lupu R, Colomer R (2005) Exogenous supplementation with omega-3 polyunsaturated fatty acid docosahexaenoic acid (DHA; 22:6n-3) synergistically enhances taxane cytotoxicity and downregulates Her-2/neu (c-erbB-2) oncogene expression in human breast cancer cells. Eur J Cancer Prev 14, 263-270.

25. Bonneterre J, Dieras V, Tubiana-Hulin M et al. (2004) Phase II multicentre randomised study of docetaxel plus epirubicin vs 5-fluorouracil plus epirubicin and cyclophosphamide in metastatic breast cancer. British Journal of Cancer 91, 1466-1471.

26. Fabian CJ, Kimler BF, Phillips TA et al. (2015) Modulation of Breast Cancer Risk Biomarkers by High Dose Omega-3 Fatty Acids: Phase II Pilot Study in Pre-menopausal Women. Cancer prevention research (Philadelphia, Pa) 8, 922-931.

27. Straka S, Lester JL, Cole RM et al. (2015) Incorporation of eicosapentaenioic and docosahexaenoic acids into breast adipose tissue of women at high risk of breast cancer: a randomized clinical trial of dietary fish and n-3 fatty acid capsules. Molecular nutrition & food research 59, 1780-1790.

28. Hooper L, Thompson RL, Harrison RA et al. (2006) Risks and benefits of omega 3 fats for mortality, cardiovascular disease, and cancer: systematic review. Bmj 332, 752-760.

29. Colomer R, Moreno-Nogueira JM, Garcia-Luna PP et al. (2007) N-3 fatty acids, cancer and cachexia: a systematic review of the literature. The British journal of nutrition 97, 823-831.

30. Villani AM, Crotty M, Cleland LG et al. (2013) Fish oil administration in older adults with cardiovascular disease or cardiovascular risk factors: Is there potential for adverse events? A systematic review of the literature. International Journal of Cardiology 168, 4371-4375.

31. Ghoreishi Z, Esfahani A, Djazayeri A et al. (2012) Omega-3 fatty acids are protective against paclitaxel-induced peripheral neuropathy: A randomized double-blind placebo controlled trial. BMC Cancer 12, 355.

32. Morland SL, Martins KJB, Mazurak VC (2016) n-3 polyunsaturated fatty acid supplementation during cancer chemotherapy. Journal of Nutrition & Intermediary Metabolism 5, 107-116.

33. Dowsett M, Nielsen TO, A’Hern R et al. (2011) Assessment of Ki67 in Breast Cancer: Recommendations from the International Ki67 in Breast Cancer Working Group. JNCI: Journal of the National Cancer Institute 103, 1656-1664.

34. Gerdes J, Lemke H, Baisch H et al. (1984) Cell cycle analysis of a cell proliferation-associated human nuclear antigen defined by the monoclonal antibody Ki-67. Journal of immunology (Baltimore, Md : 1950) 133, 1710-1715.

35. Thomas S, Johannes G (2000) The Ki‐67 protein: From the known and the unknown. Journal of Cellular Physiology 182, 311-322.

36. Jones RL, Salter J, A'Hern R et al. (2009) The prognostic significance of Ki67 before and after neoadjuvant chemotherapy in breast cancer. Breast Cancer Res Treat 116, 53-68.

37. Matsubara N, Mukai H, Fujii S et al. (2013) Different prognostic significance of Ki-67 change between pre- and post-neoadjuvant chemotherapy in various subtypes of breast cancer. Breast Cancer Research and Treatment 137, 203-212.

38. Watson PD, Joy PS, Nkonde C et al. (2009) Comparison of bleeding complications with omega-3 fatty acids + aspirin + clopidogrel--versus--aspirin + clopidogrel in patients with cardiovascular disease. The American journal of cardiology 104, 1052-1054.

39. Knapp HR, Reilly IA, Alessandrini P et al. (1986) In vivo indexes of platelet and vascular function during fish-oil administration in patients with atherosclerosis. The New England journal of medicine 314, 937-942.

40. Saravanan P, Davidson NC, Schmidt EB et al. (2010) Cardiovascular effects of marine omega-3 fatty acids. Lancet (London, England) 376, 540-550.

41. Lloyd-Still JD, Powers CA, Hoffman DR et al. (2006) Bioavailability and safety of a high dose of docosahexaenoic acid triacylglycerol of algal origin in cystic fibrosis patients: a randomized, controlled study. Nutrition (Burbank, Los Angeles County, Calif) 22, 36-46.

42. Paker AM, Sunness JS, Brereton NH et al. (2010) Docosahexaenoic acid therapy in peroxisomal diseases: results of a double-blind, randomized trial. Neurology 75, 826-830.

43. Hughbanks-Wheaton DK, Birch DG, Fish GE et al. (2014) Safety Assessment of Docosahexaenoic Acid in X-Linked Retinitis Pigmentosa: The 4-Year DHAX TrialDHAX Trial Biosafety. Investigative Ophthalmology & Visual Science 55, 4958-4966.

44. Davidson MH, Stein EA, Bays HE et al. (2007) Efficacy and tolerability of adding prescription omega-3 fatty acids 4 g/d to simvastatin 40 mg/d in hypertriglyceridemic patients: an 8-week, randomized, double-blind, placebo-controlled study. Clinical therapeutics 29, 1354-1367.

45. Field CJ, Ryan EA, Thomson AB et al. (1988) Dietary fat and the diabetic state alter insulin binding and the fatty acyl composition of the adipocyte plasma membrane. Biochemical Journal 253, 417-424.

46. Mazurak VC, Lien V, Field CJ et al. (2008) Long-chain polyunsaturated fat supplementation in children with low docosahexaenoic acid intakes alters immune phenotypes compared with placebo. J Pediatr Gastroenterol Nutr 46, 570-579.

47. Subedi K, Yu HM, Newell M et al. (2015) Stearidonic acid-enriched flax oil reduces the growth of human breast cancer in vitro and in vivo. Breast Cancer Res Treat 149, 17-29.

48. Lewis ED, Goruk S, Richard C et al. (2016) Feeding a diet devoid of choline to lactating rodents restricts growth and lymphocyte development in offspring. The British journal of nutrition 116, 1001-1012.

49. Field CJ, Thomson CA, Van Aerde JE et al. (2000) Lower Proportion of CD45R0+ Cells and Deficient Interleukin-10 Production by Formula-Fed Infants, Compared With Human-Fed, Is Corrected With Supplementation of Long-Chain Polyunsaturated Fatty Acids. Journal of Pediatric Gastroenterology and Nutrition 31, 291-299.

50. Field CJ, Van Aerde JE, Robinson LE et al. (2008) Effect of providing a formula supplemented with long-chain polyunsaturated fatty acids on immunity in full-term neonates. Br J Nutr 99, 91-99.

51. Gomez MF, Field CJ, Olstad DL et al. (2015) Use of micronutrient supplements among pregnant women in Alberta: results from the Alberta Pregnancy Outcomes and Nutrition (APrON) cohort. Maternal & child nutrition 11, 497-510.

## List of Abbreviations

| ALA | Alpha-linolenic acid |
| --- | --- |
| DHA | Docosahexaenoic acid |
| DRI | Daily recommended intake |
| DOX | Doxorubicin |
| ECOG | Eastern Cooperative Oncology Group |
| EPA | Eicosapentaenoic acid |
| ER | Oestrogen receptor |
| FFPE | Formalin fixed paraffin embedded |
| HER2 | Human epidermal growth receptor 2 |
| HREBA-CC | Health Research Ethics Board of Alberta, Cancer Committee |
| IHC | Immunohistochemistry |
| LA | Linoleic acid |
| LCPUFA | Long chain polyunsaturated fatty acid |
| NK | Natural killer cell |
| OA | Oleic acid |
| NNHPD | Natural and Non-prescription Health Products Directorate |
| PR | Progesterone receptor |
| RCT | Randomized control trial |
| TXT | Docetaxel |
